# Attitudes and behaviours of maternal health care providers in interactions with clients: a systematic review

**DOI:** 10.1186/s12992-015-0117-9

**Published:** 2015-08-15

**Authors:** P. Mannava, K. Durrant, J. Fisher, M. Chersich, S. Luchters

**Affiliations:** Centre for International Health, Burnet Institute, Melbourne, VIC Australia; Jean Hailes Research Unit, School of Public Health & Preventive Medicine, Monash University, Melbourne, Australia; International Centre for Reproductive Health, Department of Obstetrics and Gynaecology, Ghent University, Ghent, Belgium; Wits Reproductive Health and HIV Institute, Faculty of Health Sciences, University of Witwatersrand, Johannesburg, South Africa; Department of Epidemiology and Preventive Medicine, School of Public Health and Preventive Medicine, Monash University, Melbourne, Australia

**Keywords:** Maternal health, Low- and middle-income countries, Health workforce, Abuse and disrespect, Systematic review

## Abstract

**Background:**

High maternal mortality and morbidity persist, in large part due to inadequate access to timely and quality health care. Attitudes and behaviours of maternal health care providers (MHCPs) influence health care seeking and quality of care.

**Methods:**

Five electronic databases were searched for studies from January 1990 to December 2014. Included studies report on types or impacts of MHCP attitudes and behaviours towards their clients, or the factors influencing these attitudes and behaviours. Attitudes and behaviours mentioned in relation to HIV infection, and studies of health providers outside the formal health system, such as traditional birth attendants, were excluded.

**Findings:**

Of 967 titles and 412 abstracts screened, 125 full-text papers were reviewed and 81 included. Around two-thirds used qualitative methods and over half studied public-sector facilities. Most studies were in Africa (n = 55), followed by Asia and the Pacific (n = 17). Fifty-eight studies covered only negative attitudes or behaviours, with a minority describing positive provider behaviours, such as being caring, respectful, sympathetic and helpful. Negative attitudes and behaviours commonly entailed verbal abuse (n = 45), rudeness such as ignoring or ridiculing patients (n = 35), or neglect (n = 32). Studies also documented physical abuse towards women, absenteeism or unavailability of providers, corruption, lack of regard for privacy, poor communication, unwillingness to accommodate traditional practices, and authoritarian or frightening attitudes. These behaviours were influenced by provider workload, patients’ attitudes and behaviours, provider beliefs and prejudices, and feelings of superiority among MHCPs. Overall, negative attitudes and behaviours undermined health care seeking and affected patient well-being.

**Conclusions:**

The review documented a broad range of negative MHCP attitudes and behaviours affecting patient well-being, satisfaction with care and care seeking. Reported negative patient interactions far outweigh positive ones. The nature of the factors which influence health worker attitudes and behaviours suggests that strengthening health systems, and workforce development, including in communication and counselling skills, are important. Greater attention is required to the attitudes and behaviours of MHCPs within efforts to improve maternal health, for the sake of both women and health care providers.

**Electronic supplementary material:**

The online version of this article (doi:10.1186/s12992-015-0117-9) contains supplementary material, which is available to authorized users.

## Introduction

Despite major advances in reducing maternal mortality worldwide, the pace of progress is too slow to achieve the maternal health target of Millennium Development Goal (MDG) 5 [1–3]. An estimated 273,500 women die during, or after pregnancy and childbirth each year [1], whilst another ten million women suffer from pregnancy-related disease, disability or depression annually [4]. Most maternal mortality and morbidity occurs in low- and middle-income countries (LMICs) and is preventable [5].

Several factors hinder access to the health care services needed to avert maternal and newborn deaths and morbidity. These include cultural norms, gender discrimination and lack of a right’s based approach which emphasizes human dignity and attention to the needs of women in planning and delivering health services, inadequate knowledge of signs and symptoms of illness and services available, cost of services, lack of transport options and poor quality of care. The latter, quality of care, has recently received greater attention as a key reason for maternal mortality and morbidity remaining high in several countries despite substantial increases in coverage of maternal health services [6].

Quality of care is a multidimensional concept with no universally accepted definition [7]. Graham and colleagues argue that quality of care encompasses “clinical effectiveness, safety, and a good experience for the patient” [8, 9]. In the case of family planning and reproductive health services, Bruce defines quality of care as comprising six elements: choice of methods, information given to clients, technical competence, follow-up and continuity mechanisms, interpersonal relations, and an appropriate constellation of services [10]. Hulton et al., in relation to facility-based maternal health services, suggest quality of care is defined by effectiveness, timeliness, as well as the upholding of basic reproductive rights [7, 11]. In addition, quality is defined as comprising two components: the quality of the provision of care in relation to the service and the system, and the quality of care as experienced by users [11]. When care is deemed to be poor by the user, seeking of services is likely to be negatively impacted [12, 13].

The attitudes and behaviours of maternal health care providers (MHCPs) are an important element of quality as they influence both positively and negatively how women, and their partners and families perceive and experience maternal health care. Lack of respectful care from providers, such as doctors and midwives, may lead to dissatisfaction with the health system, diminishing the likelihood of seeking antenatal (ANC), delivery and postnatal services [14]. In addition, MHCP attitudes and behaviours might directly affect the well-being of patients and clients, and the relationship between patients and providers [14]. Moreover, negative attitudes and behaviours could undermine the quality of care and the effectiveness of maternal and infant health promotion efforts, in addition to compromising women’s essential right to dignified and respectful maternal health care [15, 16]. Taken together, the attitudes and behaviours of MHCPs are an important determinant of maternal and infant health outcomes [17, 18], and women being able to enjoy their basic rights of freedom from violence and discrimination and achievement of the highest attainable standard of physical and mental health [19, 20]. A recent statement by the World Health Organization (WHO) and the Human Reproduction Programme calls for greater attention, research and advocacy around the maltreatment of women at the time of childbirth in facilities [15].

Though several individual studies have explored provider attitudes and behaviours in LMICs, few have reviewed and synthesized these findings. Reviews to date have either focused on particular types of attitudes and behaviours such as disrespect and verbal and physical abuse [21–24] or specific time-periods, such as labour [21, 24]. A more comprehensive review of MHCP attitudes and behaviours in LMIC settings, which spans the continuum of the maternity period, will add important information. Such evidence, together with a summary of the influences on, and impacts of MHCP attitudes and behaviours, could inform policies and strategies to improve the utilization and quality of maternal health care. Applying systematic methods to review peer-reviewed literature, we aimed to identify the attitudes and behaviours of formal-sector MHCPs in LMICs towards their patients; influences on these attitudes and behaviours; and their impacts.

### Framework for analysis

As we could not locate an existing conceptual framework for exploring attitudes and behaviours of MHCPs, frameworks from related areas were used to develop a framework for this study. Firstly, a framework related to health worker performance and motivation was used, which identified several influences on performance using the following grouping: (1) health worker factors such as knowledge, skills, and motivation, (2) patient or client factors, namely demand for care and severity of illness, (3) work factors related to availability, clarity, and changes in guidelines and job aides, (4) health facility environment which encompasses factors such as workload, supervision, availability of equipment and supplies, and relations with co-workers, (5) administrative environment relating to the management of health workers, and (6) political and economic environment for human resource development [25]. Similarly, Franco and colleagues developed a framework related to health worker motivation which notes that motivation is influenced by factors operating at the individual, organizational, and health system levels, as well as by the broader cultural and community context [26]. Drawing on these two frameworks, and those developed by Bruce [10] and Hulton et al. [11] in relation to quality of care, we developed a conceptual framework to analyse and understand the connections between the findings from this review.

**Fig. 1:**
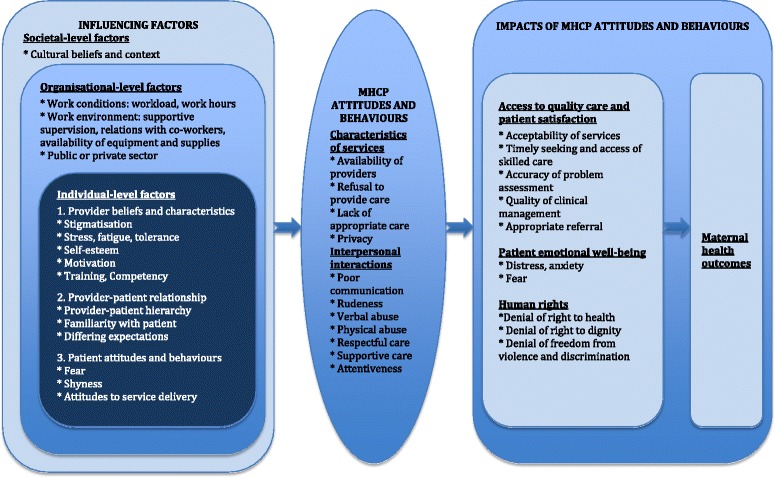
Conceptual framework: Influences on and impacts of MHCP attitudes and behaviours

The framework shows the factors that influence MHCPs’ attitudes and behaviours, the resultant types of attitudes and behaviours and their corresponding effects (Fig. 1). Determinants at the: (1) individual-level such as provider beliefs and characteristics, provider-patient relationship, as well as patient’s attributes, attitudes and behaviours; (2) organisational-level such as work load and working environment including supportive supervision, relations with co-workers and availability of medicines and commodities; and (3) societal-level namely cultural beliefs, shape positive and negative attitudes and behaviours of health workers. These attitudes and behaviours, in turn, impact on the patient’s emotional well-being, satisfaction with care, and access to services – all of which are also interrelated. By having an effect on these elements, which determine quality of care, attitudes and behaviours ultimately influence maternal health outcomes.

## Methods

### Search strategy

Five electronic databases were searched: the Cochrane Library, CINAHL Complete, Medline (PubMed), Popline and PsychInfo. Search strings were developed based on identifying key words and medical subject headings related to the population (MHCPs in LMICs), the “intervention” (attitudes and behaviours), and potential outcomes (satisfaction, acceptability, access, utilization, and health-seeking behaviours). The full search strategy is included as Additional file 1. Reference lists of included studies and reviews located on the topic were examined to identify additional literature. Retrieved records were imported into the reference management software EndNote X4 and assessed against inclusion and exclusion criteria in three stages - screening of titles, abstracts, and finally full texts.

### Inclusion and exclusion criteria

This study was limited to literature published in English from January 1990 to 1 December 2014. As the aim was to explore the breadth of the research undertaken on MHCP attitudes and behaviours in LMICs, all types of study design were included. MHCPs were defined as trained providers (such as medical doctors, nurses, midwives and paramedics) delivering antenatal, abortion, childbirth or postnatal services (including family planning) up to one year after childbirth. Studies on experiences of HIV-positive women within maternal health services were not included here as HIV itself incurs marked stigma and discrimination, with corresponding implications for service utilization and health outcomes [27–33]. Given that provider attitudes and behaviours towards HIV likely differ considerably from other conditions, this was considered a separate review and beyond the scope of this study. The LMICs included were drawn from the World Bank’s classification of countries’ income status in July 2012.

Studies were included if they reported on the types of attitudes and behaviours, the factors influencing these, and/or the impacts resulting from certain attitudes and behaviours. Reports which simply stated that the attitude or behaviour was ‘positive’ or ‘negative’ without providing additional details on the type of attitude or behaviour, or the influences or impacts of the positive or negative attitudes and behaviours were excluded. We also excluded studies related to health care for children; case studies of the experience of one patient or one MHCP only; and studies describing factors which influence quality of care without specifying the impact of MHCP behaviours and attitudes.

### Analysis

A thematic analysis approach was used to synthesize the evidence located. Text relevant to attitudes and behaviours, and their influences and impacts, was extracted from full-text documents and those that were similar or conceptually-related were grouped together. Thus, for example, insulting and humiliating speech, shouting and scolding were classified as ‘verbal abuse’; whilst ignoring patients or being uncaring, dismissive or hostile were classified as ‘rudeness’. Selected quotations from participants as reported in the studies were copied verbatim to further illustrate dominant themes or notable exceptions to these.

For each paper included in the review, information was extracted into a standardized data tool on: (1) study characteristics (first author and year of publication, study design and setting); (2) study population; (3) type of facility (public or private) and health worker cadre; (4) type of attitude or behaviour, grouped as positive and negative; (5) factors influencing attitudes and behaviours; and (6) impact of attitudes and behaviours.

## Results

**Fig. 2:**
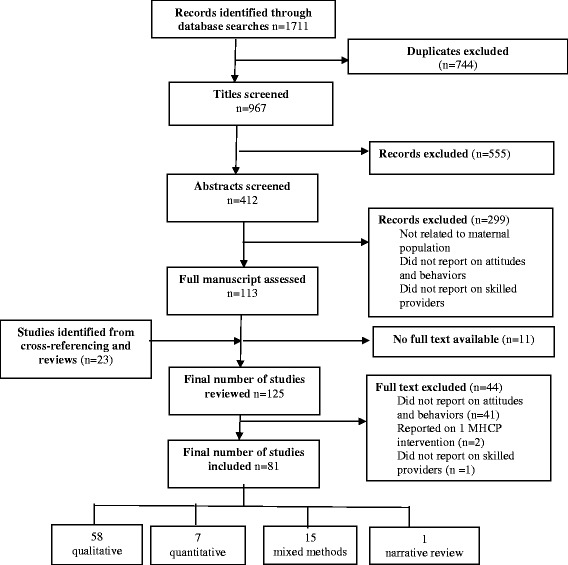
Flowchart of different stages of the systematic review

Of the 967 titles and 412 abstracts screened, 125 full text papers were obtained and reviewed, and 81 studies included in the review (Fig. 2). Almost all of the 44 papers excluded on full text did not provide information on MHCP attitudes and behaviours (n = 41), two described experiences with one MHCP only, and one paper reported on the attitudes of providers who were not skilled.

### Included studies

Most included studies, 58, used qualitative research methods (Additional file 2: Table S1). An additional 15 studies used mixed qualitative and quantitative methods, seven were quantitative surveys, and one was a narrative review. Of included studies, none evaluated interventions that aimed to alter MHCP attitudes or behaviours. Close to two-thirds of the papers (n = 48) explored attitudes and behaviours from patient or community perspectives only. The remainder reported health care provider perspectives only (n = 4), these together with individual patient or community perspectives (n = 23), a mixture of provider, patient/community, and researcher observations (n = 4) or the latter two only (n = 2). The most common regional setting was Africa (n = 55) followed by Asia and the Pacific (n = 17), Latin America (n = 10) and the Middle East (n = 2). Four papers were set in more than one country. Of the 77 single-country studies, nine were from Tanzania, seven from South Africa, six from Nigeria, five from Uganda, and four from Kenya.

Fifty-five studies provided evidence on the impact of attitudes and behaviours, while forty described influences on attitudes and behaviours. All studies apart from one, reported on types of attitudes and behaviours – negative (such as verbal and physical abuse) and positive (such as being friendly and respectful). Authors most commonly focused only on negative attitudes and behaviours (n = 58), with 20 describing both negative and positive ones. The attitudes and behaviours of health care providers working in public facilities only were examined in 46 studies, and those of health care providers working in both public and private facilities were examined in another 13 studies. The majority of publications did not specify the cadre of health care provider studied (n = 46); while 34 articles provided evidence of the attitudes and behaviours of nurses, 33 of doctors and 32 of midwives. Attitudes and behaviours were primarily reported on at the time of childbirth (n = 66), followed by during the antenatal period (n = 30), family planning consultations (n = 6), the postnatal period (n = 4) and at abortion (n = 3). Study findings are presented below, disaggregated into positive and negative attitudes and behaviours.

### Positive MHCP attitudes and behaviours

#### Types of positive attitudes and behaviours

Twenty-three studies, the majority of which were set in Africa (n = 17, 31 % of studies from the region), reported on a range of positive attitudes and behaviours of MHCP [34–50], mainly at the time of delivery (n = 16) and during antenatal care (n = 8). Most commonly in these reports patients described MHCPs – working in public and private facilities – as being caring when women were seeking ANC [21, 22, 29, 34], in labour [20, 25, 27–29, 34, 36, 39, 41–43, 45, 48, 51–54]), or having an abortion [31]. For example, a mother in Bangladesh noted, by “*continually checking up on their [women’s] conditions, providing medications and regularly asking after their health*” [34]. Encouragement and support during childbirth was another recurring theme highlighted in five studies [34, 35, 42–44]; in the words of one mother: “*During the delivery, the support of the doctor was very important to me. He was very kind and humane. I will never forget his encouragement*” [Lebanon] [43]. Women also mentioned respect and having been treated well by providers [34, 38, 40, 46].

In a few studies, MHCPs were reported as being friendly [42, 51], kind [44, 45] and sympathetic [37, 39]. Providers were also described as polite [38, 40, 46, 50], welcoming [38, 41], informative [38, 43], helpful [39, 40] and attentive [55].

In a survey in Lusaka, Zambia exploring access to and quality of maternity care, just over half the 845 women who had delivered in a health facility praised midwives for ‘good personal treatment’ of maternity patients [44]. Of the 821 reflections provided by these women on MHCP attributes that were valued and remembered, close to half related to ‘kindness’ and ‘encouragement’ [44]. Another study also highlighted exceptional instances of generosity from MHCPs in Argentina, where doctors had paid for maternal health services unaffordable to patients [46].

A survey in Tanzania found differences in the interpersonal aspects of care between public and private facilities. Of women attending public facilities (n = 166), 93 % reported that providers showed interest, 70 % were not interrupted by providers during conversations, 98 % felt providers were polite and 71 % were asked about their concerns. For women attending private facilities (n = 188), similar proportions noted that providers showed interest and were polite (95 % and 98 % respectively), while more had not been interrupted during conversations (87 %) and were asked about their concerns (81 %) [38]. These differences were statistically significant (*P* < 0.001 for non-interruption of conversations, and *P* = 0.02 for asking about concerns). In South Africa, a mixed-method study found differences in behaviour based on the location of the facility: a higher number of women receiving services in two urban sub-district public obstetric facilities reported respectful behaviour from health workers as opposed to women from rural facilities (63 % and 66 % for rural versus 75 % and 72 % for urban, *P* < 0.01) [56].

### Factors influencing positive attitudes and behaviours

Five studies reported reasons for the positive attitudes and behaviours of MHCPs. In Bangladesh, the understanding and caring nature of providers in private facilities was attributed, by the study researchers, to the providers familiarity with patients’ cultural practices and communities [34]. In a similar vein, MHCPs working in public and private facilities in a few countries in Africa, as well as in the Dominican Republic, were more likely to show positive attitudes and behaviours when the patient was from the same catchment area as the heath facility [38] or when the patient was known to them [57–59]. As stated by one study participant, “*Doctors and nurses only pay attention to their friends and relatives*” [Mothers, Nigeria] [57], whilst authors of another study undertaken in Ghana, Kenya and Malawi remarked: “*At health facilities, communication tended to be more two-way if a woman…had a familial relationship or friendship with the health worker*” [59].

### Impacts of positive attitudes and behaviours

In nine studies, the presence of MHCPs who were respectful, caring, friendly, helpful or sympathetic were important factors in encouraging demand for maternal health care, including antenatal care [55] and facility-based delivery [37, 39, 43, 47–49]. In a survey of 178 women across four sites in South Africa, 11–15 % of respondents cited friendliness of staff as a reason for attending antenatal care [49]. These experiences meant clients were more likely to be satisfied with quality of care [39, 43, 48], and feel positive emotions, such as higher self-esteem [43]. For example, one woman in relation to ANC consultations with an obstetrician, said: “*When I visit her I feel relaxed, I feel less pain because I like her. She asks me about my problems, I tell her and she answers to all my questions. She talks about everything and she explains everything*” [Mother, Lebanon] [43]. One study found that women experiencing positive attitudes and behaviours were more likely to decide to return to a facility than those experiencing negative ones. A positive attitude of one MHCP even compensated for other negative experiences, with one woman remarking: *"I will go there again, because even though one of the nurses was unfriendly and impatient, the other was very accommodating and I pray I will meet someone like her anytime I have to go there*” [Mother, Ghana] [39]. Lastly, one study rated quality of care higher when MHCPs were attentive, polite and showed interest in patient’s concerns [38].

### Negative MHCP attitudes and behaviours

#### Types of negative attitudes and behaviours

Negative attitudes and behaviours were clustered into two areas. Firstly, negative interpersonal interactions between providers and patient, which encompassed verbal abuse or inappropriate communication, and physical abuse. Secondly, negative behaviours of providers in terms of actual service delivery, which manifested as deficiencies in availability of services, lack of privacy during patient care and unwillingness of providers to accommodate traditional practices.

##### Interpersonal interactions between provider and patient

The most commonly reported negative behaviour (n = 45) was verbal abuse during ANC (n = 12) and childbirth (n = 35) – specifically shouting, scolding or use of insulting language [34–37, 42–44, 48, 51, 57, 59–80]. Two surveys on birth care undertaken in Zambia and Tanzania found that shouting and scolding was the commonest complaint related to MHCP attitudes and behaviours, reported by 56 % (of 845) of women sampled in Zambia and 8.7 % (of 153) of women in Tanzania [44, 81]. Only one study each reported verbal abuse during postnatal care [55], at the time of abortion [64] and when seeking family planning services [48]. Many studies providing evidence on verbal abuse sampled public sector facilities (n = 43), whilst nine studies also noted instances of this behaviour in private facilities. Evidence from Ghana specifically indicated that verbal abuse is more problematic in public than private facilities [66]. The majority of studies reporting on verbal abuse were set in Africa (n = 34, 62 % of studies from the region), with fewer in Asia (n = 6, 35 % of studies from the region), Latin America (n = 4, 40 % of studies from the region) or the Middle East (n = 1, 50 % of studies from the region). Though midwives (n = 19) were most commonly cited as being verbally abusive, a similar number of studies (n = 21) also did not specify the type of health worker.

Thirty-five studies described rude behaviour from MHCPs during all stages of seeking maternal care (antenatal, delivery and postnatal), with all these papers documenting examples from public health facilities [34–37, 39–42, 46, 49, 51, 58, 60–64, 71, 82–92] and very few from private ones [39, 66, 81, 93]. Most studies did not pinpoint the cadre of health worker who was rude (n = 23), and were set in Africa (n = 31, 56 % of studies from the region) and Asia (n = 10, 59 % of studies from the region). In Bangladesh, Benin, Ghana, Nigeria, Tanzania and South Africa, studies recounted how providers ignored, dismissed or ridiculed the opinions of women when they expressed their needs or voiced their opinions [34, 35, 41, 65, 66]. One pregnant woman in South Africa explained how a nurse had discounted her opinion: “*If you air your views or your opinion, they laugh at you and ridicule you*” [65]. Anger, and hostile or impersonal behaviour from nurses and midwives was another recurring theme [41, 51, 67]. Specific instances of these behaviours included when assistance was requested by patients [39], or when postnatal services were sought at facilities other than where delivery had taken place [60]. Other commonly reported experiences of MHCPs were harsh and condescending attitudes [34, 35, 82, 84, 87, 94, 95], and a lack of sympathy [39, 42, 63, 84]. In another study set in South Africa, women who had experienced stillbirths complained about health workers’ lack of sensitivity in placing them in wards together with women and their live babies [56]. In the words of a woman who had a stillbirth, “*I could have been better off if they took me to a room for the mentally ill people rather than in a room where there were people carrying their babies and I stayed there and I was crying cause babies were crying and I could not take it you know”* [56].

In other instances, patients and providers themselves described MHCPs as authoritarian and frightening [70, 91, 92], particularly during childbirth [70, 91, 92]. In one study in Mexico, for example, researchers remarked in relation to application of an epidural block: *“In this particular case, the doctors used intimidation as a strategy to keep the women immobile” “if you move, you'll be responsible if we prick your baby”, “if anything happens to the baby, it will be your fault”* [92]. A mother recruited in a qualitative study in the Philippines remarked, *“…the doctor was mad at me when I told her that the baby is about to come out. She told me to hold on from pushing or else she will suture me inside there”* [96]. During ANC, researchers of one study observed that pregnant women were ordered to undertake actions – such as for collection of blood specimens– in an authoritarian manner [97].

An overall lack of communication from MHCPs was reported in 16 studies [34, 41, 43, 45, 56, 58, 60, 64, 68, 77, 80, 94, 95, 97, 98, 106], primarily in public facilities (n = 13) with doctors (n = 8) and nurses (n = 8) most commonly cited in the evidence, though nine studies also referred to ‘health workers’ more generally. One study, which specifically looked at communication to young pregnant women (ages 14 to 20 years) with complications, found that doctors and midwives did not provide important information on how complications might affect the baby or why tests to monitor complications were being performed [97]. In addition, patients were not given the opportunity to clarify doubts or ask questions [97]. In other studies, information was not provided about abortion care [64], progress of labour [34, 43, 58], the health and sex of the baby [34], as well as safe neonatal care practices [34]. In certain cases, patients also did not know the reasons for, or outcomes of physical examinations [58, 60, 68], medication [77, 98], and surgical procedures, such as caesarean sections [80]. In two studies, one exploring communication during ANC and another on women who experienced stillbirths, women reported learning about health outcomes through overhearing conversations between health workers rather than being told directly [56, 97].

Seventeen studies included accounts of physical abuse from MHCPs, mainly during or after childbirth [37, 39, 41–42, 48, 53, 60, 62, 63, 68, 69, 74–77, 79, 81] – most of which were set in Africa (n = 13) and cited midwives as being abusive (n = 7). Women were beaten, slapped or had their hair pulled when they were perceived as not following instructions or not pushing during labour [37, 41, 42, 60, 74, 77]. A mother who participated in a study in Benin said: “*They asked why I could not stay still to give birth, and they started to beat me*” [41]. In a survey undertaken among 1,779 women in Tanzania, two women reported being sexually harassed and 4 women reported rape [81].

##### Characteristics of the health services delivered

This section reports on provider neglect or abandonment of patients, limited availability or absenteeism, and refusal to deliver services. The theme of neglect or abandonment recurred frequently, reported in 33 studies [35–37, 40, 42–44, 58, 60, 61, 63, 64, 68–70, 74, 75, 80, 81, 85, 87, 88, 95, 98–107] – again primarily in government run hospitals and centres (n = 30). Neglect or abandonment was mainly cited in studies set in Africa (n = 22, 40 % of studies from the region) and Asia (n = 10, 59 % of studies from the region), and demonstrated by nurses (n = 17) and doctors (n = 12), or by facility health workers in general (n = 16). Several studies provided accounts of women being abandoned during consultations or in critical situations when assistance was required [36, 40, 58, 68, 74, 80, 85, 95, 99, 105]. A common experience described in study reports was being left alone in the labour room during childbirth without any supervision, or delayed attendance, because nurses and midwives were sleeping, chatting, watching television or did not inform doctors of the delivery [43, 58, 60, 68–70, 74, 75, 80, 85, 88, 95, 98, 100, 106]. Researchers of a quality of care study in the Dominican Republic noted that in a labour ward of a referral level hospital: “*At one point a woman gave birth unattended while a group of students stood around the bed across the aisle from her, no one noticed the very clear sounds of impending delivery amid the noise, cries, and conversations*” [68]. In another study, a mother in Tanzania shared her experience of neglect: *“(…) they placed me on a labour bed, and they just sat there chatting, when you yell with pain, they say you just wait, shouting from where they were, “you are not yet ready for delivery”, so I kept on waiting while being tortured with pain*” [85]. In a Zambian survey of health facility-based birth care, 16.5 % of women sampled (n = 845) stated that the health worker simply “did not come” [44]. Similarly, in another quantitative study undertaken in Tanzania, 8 % and 4 % of 1,779 women surveyed reported being ignored when needing help and delivery without an attendant respectively [81]. Lastly, women who had undergone abortions in a Vietnamese study reported being left alone in the recovery room [64].

Lack of availability or absenteeism of MHCPs was mentioned by participants in six studies [57, 67, 71, 72, 90, 106] in public and private facilities; in the words of one mother in Malawi: “*I went to (…) health center, and the health worker was not there…*” [106]. In a qualitative study in West Java, Indonesia, exploring the reasons why women delivering at home choose either the trained village midwife or the traditional midwife, the researchers heard complaints of absenteeism and being left alone during labour by trained midwives: “*They say the traditional birth attendants are more patient…This attitude is different from midwives. Sometimes after the physical examination, the midwife leaves if she thinks it is not the time for delivery yet. In contrast, the traditional birth attendant will wait patiently and accompany the woman all along.”* [traditional birth attendant, Sukabumi] [90]. Potential bias with this finding reported in the study must be acknowledged however, given that it was illustrated by an example provided by a traditional birth attendant (a ‘competing’ provider) and not cared for by a midwife.

Studies from Africa and Asia (n = 1) reported doctors, nurses and midwives refusing to provide care or treatment [51, 56, 63, 77, 79, 101]. In other examples, patients were forced to clean up after themselves following childbirth [39, 42, 62, 68, 69], refused assistance to get up or use toilet facilities [42, 99], or denied pain medication at the time of abortion [79]. Researchers following childbirth in a public hospital in the Dominican Republic also observed: “*Women brought their own towels and clothes and would often get themselves up, dry themselves off with their own towels, and change from their wet, bloody clothes (if they weren’t already naked) into their own night clothes. They then walked barefoot across the bloody, slippery floor to the wheelchair*” [68].

Fourteen studies reported that doctors (n = 9), nurses (n = 5), midwives (n = 4), and general health staff (n = 6) sought bribes to provide any care or better quality care [35, 46, 58, 61, 76, 77, 81, 84, 86, 95, 98–100, 103], primarily in government run facilities (n = 10). A mother in a study in Afghanistan reported: “*After the operation I needed a bed pan, but they gave it only after I offered them some money!*” [95].

Women in five studies from Asia, Africa, and Latin America, expressed discontent with MHCPs’ working in public and private facilities lack of willingness to accommodate traditional practices during childbirth, such as applying butter on the abdomen [88], allowing delivery in the traditional and preferred position of squatting or kneeling [34, 78, 84], and giving the placenta to families following childbirth [61, 78, 88]. In a study undertaken in Guatemala, a mother described how hospital staff refused delivery in the kneeling position: *“Ah! I wanted to have the baby kneeling because I had become used to having my babies kneeling . . . I had told them there at the hospital that I wanted to get down [from the stretcher] and have it kneeling down because when kneeling I can feel when it’s coming, but I couldn’t, they scolded me there…”* [78].

In nine studies set in countries of Africa, Asia, Latin America and the Middle East, doctors, midwives and nurses were said to be impatient and made women feel rushed during the process of childbirth [39, 43, 52,68, 84, 90, 99, 102, 104]. Researchers heard that health care providers often opted for episiotomy or surgery to deliver the child quickly [84, 102]. In a study undertaken in Tanzania, a pregnant woman commented: *“…they never wait to see whether you can deliver normally, but they hurry in doing an operation on you”* [104].

MHCPs’, namely nurses’ (n = 5), doctors’ (n = 4), and midwives’ (n = 1), lack of regard for privacy was a concern raised by women in ten studies across Africa (n = 4, 7 % of studies from the region), Asia (n = 4, 24 % of studies from the region), Latin America (n = 1, 10 % of studies from the region) and the Middle East (n = 1, 50 % of studies from the region). In Ghana and Zimbabwe, participants expressed displeasure with nurses conducting interviews in a loud voice or undertaking examinations in open settings, such that other patients could hear or see [66, 97]. As remarked by a participant in the Zimbabwe study: “*As for that place (reception area), everybody is sitting there and looking at each other. You cannot talk about all your concerns. The kind of sickness that brought you there, you cannot say it before other people. If you want to talk about how your sickness started, it is not easy to say everything in front of others. You feel that they are listening*” [97]. In Tanzania, close to 3.5 % of 1,779 women surveyed reported a lack of physical privacy during childbirth in public and private facilities [81]. Similarly, in other countries in Africa as well as in Asia and the Caribbean, women felt that their privacy was not respected during examinations prior to or following abortion [64], or at the time of childbirth, with many health facility staff allowed to enter and leave the room [34, 43, 60, 64, 68, 88]. A study which looked at differences between women admitted in two urban and rural sub-district obstetric facilities found that a significantly higher proportion of participants reported respect for privacy in urban facilities – 95 % and 89 % of women surveyed as opposed to 86 % and 89 % for rural facilities (*P* < 0.01) [56].

### Factors influencing negative attitudes and behaviours

In contrast to the limited data available on factors determining positive attitudes and behaviours, there was substantial evidence from 29 studies of influences on MHCPs’ negative attitudes and behaviours, based on provider (n = 20) and client (n = 12) perspectives, as well as the observations of researchers and statistical analyses (n = 10).

#### Organization-level factors

Deficiencies in MHCPs’ work conditions and working environment were widely reported (in 27 studies) as accounting for negative attitudes and behaviours, by both providers and clients in evidence from countries of Africa, Asia, Latin America and the Middle East. Heavy workloads and long working hours [34, 35, 42, 51, 67, 68, 77, 94, 106], weak supportive supervision or poor relations with co-workers [35, 42, 57, 64, 77], insufficient salaries [51, 56, 57, 74, 77, 86] and a lack of equipment and supplies to deliver the services required [64, 90] were common, mostly in public facilities. No important differences in patterns of the evidence on work-related factors were seen between geographical area or cadre of health worker, except for workload which was more commonly reported in African studies (n = 10 or 18 % of studies from the region versus ≤ 2 studies from other regions) and by midwives and nurses (n = 7 and n = 6 respectively versus n = 4 for doctors), as well as insufficient salaries, which was mainly cited as a factor in African studies (n = 4, 7 % of studies from the region versus n = 1 from Asia, 6 % of studies from the region).

Deficiencies in working conditions and the work environment, in turn, resulted in stress, fatigue, frustration and poor job satisfaction for MHCPs [41, 42, 51, 57, 85, 86] leading to poor communication and uncaring attitudes towards patients [76]. One provider in a multi-country study in Africa noted: “*If the colleague is struggling to meet some costs and his work is heavy and the roof of his house is leaking, then all of this will play on his work. Someone who is angry all the time about things that are out of reach….this person can pour his anger on the patients! He will not greet kindly. He does not even care whether the treatment has any effect. At this time he does not even want to work”* [76]. Poor communication and rude behaviours were also attributed to inadequate training in six studies [34, 64, 83, 84, 89, 108], and poor remuneration cited as a reason for seeking bribes in public and private facilities in a multi-country study set in Burkina Faso, Ghana, and Tanzania, and in studies from Afghanistan, and Pakistan [76, 77, 86]. One paper highlighted lack of space at facilities as a reason for inability to provide privacy [109]. Importantly, another study found that impersonal attitudes among MHCPs stemmed from their frequent encounters with sickness and death [67]. A few studies reported that MHCPs refused to provide services to patients due to fatigue [63], already having seen the number of patients allowed under the daily quotas for consultations with new patients [42], and patients being referred from traditional birth attendants [51, 61] or doctors [100] as the MHCPs (doctors, midwives, and nurses) were disapproving of other providers.

#### Individual-level factors

##### Provider beliefs and characteristics

Fourteen studies, nine set in Africa (16 % of studies from the region) and 5 in Asia (29 % of studies from the region), found that MHCPs working in public and private settings held prejudices towards certain patient attributes, such as socio-economic status, education level and ethnicity. This resulted in discrimination or rude behaviours towards poorer, less educated, and rural-dwelling patients, or those belonging to ethnic minorities [34, 59, 60, 81, 82, 91, 93, 106, 108]. Provider beliefs related to age and marital status norms for childbearing, as well as towards termination of pregnancies, also influenced behaviours, with midwives and doctors for example, showing disrespect towards pregnant women of older ages or women undergoing abortion [61, 79]. In a Zimbabwean study, a lack of communication from ANC providers seemed to be linked to the young (14 – 20 years) age of pregnant women: *“A 14-year-old girl said that she was frustrated by midwives who just looked at her but had nothing to say to her. Instead, they talked to her mother who had accompanied her”* [97].

In some instances, women deemed as being ‘socially deviant’, for example teenage mothers, were reported to have been verbally abused, mocked, or not cared for as well as other women [42, 61]. A review described how physical abuse, in the form of denial of pain medication to abortion patients, stemmed from provider beliefs and prejudices: “*I don’t spare these young girls who become pregnant. They should be made to feel the worst pain so that they can fear having sex aimlessly”* [Doctor, Kenya] [79]. In a study in Timor Leste, a facility manager remarked that midwives were more likely to get angry at women who were primiparous and didn’t have ‘experience’ with childbirth, as the women might not be able to push when directed [54]. Finally, four papers suggested that prejudices of MHCPs towards traditional practices meant that MHCPs refused to accommodate traditional practices at the time of childbirth [34, 41, 69, 84].

Patients from African and Asian settings (n = 4, 7 % of studies from Africa and n = 1, 6 % of studies from Asia), sometimes remarked that characteristics of midwives and nurses themselves, such as age and marital status, influenced behaviours and attitudes. Older and married providers were commonly described as more understanding, mature and caring [35, 41, 76, 90, 108]. For example, one study participant explained that: “…*Those who were there that day seemed to be young midwives, women who have never had a child. There’s always a difference between a young midwife and an older one. Older midwives would have known how they used to treat women at the maternity hospital when they were giving birth compared to how they treat them now*” [Mother, Benin] [41]. One study found that gender norms within society and at the workplace dictated interactions between female providers and their patients. Lady Health Workers and Lady Health Visitors in Pakistan reportedly were harsh and strict towards patients (whom include men, for example during family planning counselling sessions) so as to avoid being perceived as ‘open’ and ‘friendly’, which due to cultural norms, could lead to being interpreted by men and other women in the community as ‘easy’ or “sexually loose” for men: *“People make scandals very quickly. Even if you just smile at a patient, they become suspicious of your character”* [Lady health worker, Pakistan] [86].

##### Provider-patient relationship

Another factor commonly reported as a reason for less respectful treatment of women in countries of Africa (n = 3, 5 % of studies from the region) and Asia (n = 3, 8 % of studies from the region) was the belief by doctors, nurses and other health care providers that they are of higher social status than patients [34, 42, 64, 76, 77, 97]. In a study in Afghanistan, for example, women were expected to accept doctors’ prescriptions without requiring any further explanation or information due to their lower status in society [77]. Hierarchy differentials also affected communication with patients, as remarked by one pregnant woman in a Zimbabwean study: *“He talks to his friends, the staff, but not me*” [97]. Similarly, in South Africa and Viet Nam, rude and abusive behaviours towards patients seemed to enable MHCPs to feel superior and maintain their middle-class and educated identity [42, 64]. One Vietnamese doctor described: *“…I sometimes disappear for a quarter or half an hour. Indeed, I have nothing to do, but that is the way we* (health staff) *let them know who is superior here”* [64]. In one study, MHCPs assigned higher priority to personal commitments, refusing to provide services to patients in order attend to personal matters [76].

In another study, providers justified being authoritarian or frightening in order to instil obedience in patients, which, in turn, they believed ensured safer delivery: “*At times the midwife must get angry and threaten the woman, not abuse or beat her, but tell her to obey in order for the baby to be delivered safely. Otherwise, I tell her, she may return to the house without her baby*” [Midwife, Mozambique] [91].

##### Patient attitudes and behaviours

Frustration with patient behaviours and attitudes was widely reported as giving rise to negative reactions, such as verbal abuse, among MHCPs, mainly in African public sector settings. Doctors, nurses, and midwives complained about women and their families presenting late for ANC or delivery [41, 42, 57, 64, 69, 76, 108], not complying with medical advice [64] including delivering at home [55], or falsely accusing providers of mistreatment [91]. One auxiliary midwife in Burkina Faso explained: “*These women insist to try and deliver at home. This is something that we discuss at village meetings. Yet still it happens. They only come here when things go wrong. In such cases I do not hesitate to scold them*” [76]. Other specific examples of triggers of verbal abuse were when women had not followed instructions regarding attendance at ANC [36, 59, 62, 65, 66, 73, 109], did not possess an ANC card [71], had many previous pregnancies [36, 107, 109], or were teenage mothers [36, 42, 109]. One study even found that patients were denied care or treatment by MHCPs if in the early stages of pregnancy [101].

Attendants shouting at and scolding women at the time of childbirth appeared common across all regions when pregnant women had difficulties pushing, or wanted to deliver in a traditional position, such as kneeling [34, 35, 37, 42, 43, 60, 62, 66, 68–70, 77, 78]. Authors of a study in Bangladesh described a health centre worker screaming at a woman during childbirth: “*You village woman, don’t you know the rules for delivering a baby? Push down when you feel cramps in your stomach*” [34]. Abuse also sometimes followed women not wearing convenient clothing or not washing prior to attending a health facility [109]. Women were sometimes insulted for speaking softly, walking into the wrong consultation room, or for ‘causing chaos’ in corridors [42, 64]. Verbal abuse was more likely when mothers tried to seek attention, assert their rights, or contradicted midwives’ opinions [42].

Regression analyses of data collected from a survey in Tanzania found that the odds of abuse and disrespect during childbirth were higher when women were primiparous (odds ratio (OR) = 1.26, *P* < 0.05), had reported a ‘low’ mood in the previous 12 months (OR = 1.27, *P* < 0.05), had a history of rape or physical abuse (OR = 2.29, *P* ≤ 0.001), and reported complications during delivery (OR = 1.69, *P* ≤ 0.001) [81]. Women who underwent caesarean sections on the other hand, were less likely to report disrespectful treatment (OR = 0.66, *P* ≤ 0.01), with the authors suggesting that MHCPs were more careful and respectful as a result of performing a surgical procedure [81].

### Impacts of negative attitudes and behaviours

Two-thirds of all papers included in the review reported on the impacts of negative MHCP attitudes and behaviours. These impacts affected four key domains: emotional well-being, client satisfaction with care, overall access to quality services and maternal health outcomes.

#### Patient’s emotional well-being

In seven studies, rude behaviours, poor communication, as well as verbal and physical abuse from MHCPs were found to result in distress and fear among patients in Africa (n = 4, 7 % of studies from the region) and Asia (n = 3, 18 % of studies from the region) [34, 42, 70, 77, 82, 107], or an absence of trust in providers [41]. All these factors reportedly affected care-seeking behaviours and undermined patient-provider interactions. An Afghan doctor in relation to negative MHCP attitudes and behaviours commented: *“I am sad to say that patients are afraid of us, they do not dare to ask questions”* [77]. Two studies found that such behaviours also made women feel like ‘passive objects’ during childbirth, with no control or engagement in the experience [34, 83]. One of these studies, undertaken in Bangladesh, provided an account of the emotional state of a patient and her family at the time of childbirth: “*The woman was lying on the bed, looking anxious. Without informing her, a nurse removed the sari from her abdomen and examined her body. The woman’s mother was also in the labour room and asked about foetal movement. The nurse did not respond to the mother’s question at first, she finished the examination and then said, “The baby is all right.”…Later that night, when she was taken to the labour room again, the woman still looked anxious. She lay down on the labour bed and they started intravenous fluid without giving her any explanation. Then a nurse examined her vaginally, without informing her why or what she found. Continuous monitoring was going on but the family was not informed about the progress of labour. The woman’s mother [who was now outside the labour room] became very upset and started crying”* [34].

#### Access to quality care and patient satisfaction

In Ghana and Nigeria, women reported low satisfaction with maternal health care due to physical abuse and rude behaviour from MHCPs [39, 67]. One participant commented: *"The services were not so good, the attendant … refused when I needed to hold her while I was in pain she said it won't change anything…even when I asked the ward assistant for water she brought me chilled water, when I said I preferred tap water, she became angry"* [Mother, Ghana] [39]. Binary logistic regression of results from a survey in Ghana based on the Picker questionnaire^1^ found that women who were only sometimes treated with respect by MHCPs were 3.6 times more likely to be dissatisfied with childbirth care than those who were always treated with respect [110].

Several studies in different settings demonstrated that MHCPs’ poor attitudes and behaviours, or perceptions of them, were important barriers to seeking antenatal care and facility delivery in Africa, Asia and Latin America [41, 42, 48, 49, 57, 59, 60, 62, 66, 67, 71, 77, 78, 85, 89, 90, 100, 101, 104]. Many women did not attend ANC because of poor communication and disrespectful treatment by providers [38, 41, 48, 49]. In The Gambia, one midwife narrated: *“She* [patient] *was vomiting throughout the night, the following morning the husband decided to take her to the health centre but she refused… … …she has not yet got an antenatal care card. She feared the nurses because if she goes to complain about the vomiting she will be asked the card and without it they* [nurses] *will tell her all salty words. She may be insulted or may even not be given medicine"* [71]. In certain African settings, women attended ANC only to obtain an ANC card, which was necessary in order to book deliveries [39, 63, 71, 101, 106] or out of fear of being abused by MHCPs for not attending ANC [71]. For example, five of 83 women (6 %) surveyed in Mozambique stated that they only presented for ANC to obtain a prenatal evaluation form and vaccination record card as proof of attendance so that they would be admitted to the maternity clinic at the time of delivery. Otherwise, these women saw no benefit in attending ANC, largely due to the attitudes and behaviours of the personnel at the maternity clinic [101].

Twenty-four studies in various African and Asian settings, as well as one study from Latin America, stated that negative attitudes and behaviours were a barrier to facility-based delivery [34, 36, 37, 51, 57, 60–63, 65, 72, 74, 82, 84, 85, 87, 88, 98, 99, 103, 105, 107], with women preferring home delivery with traditional birth attendants [51, 88, 90, 102, 103]. In a Ugandan study, rude staff was the most common reason cited for women feeling uneasy about delivering at a health centre [36]. Delivery at hospitals was viewed as a last resort, even in the case of high-risk deliveries or complications during labour [84]. One mother in a Ugandan study remarked that, despite the shortfalls in the medical capacity of traditional birth attendants and family members, at least with them: *“Nobody will restrain/rebuke you and sometimes the attendant will sympathetically cry along with you”* [84]. A study specifically examining the experience of adolescents with ANC and delivery services in Uganda, found that pregnant adolescents sought ‘safety and empathy’ from health workers [37]. Being neglected, and verbally and physically abused by MHCPs instead, therefore served as a deterrent to seeking facility-based ANC and delivery [37]. In Guatemala, verbal abuse from staff was a reason given for why pregnant women and their families did not attend maternity waiting homes [78], whilst in Zimbabwe such behaviours discouraged women and their families from accepting referrals to hospitals [62]. In Bangladesh, MHCPs’ lack of willingness to accommodate traditional delivery positions was a deterrent to delivery at a health centre, as noted by one mother: “*I can’t even think about giving birth lying down on the bed. How is it possible? How do women push down in this position? I don’t think 1 would be able to deliver at the BHC (health centre)!*” [34].

In a study exploring pain management in abortion, a midwife in Kenya explained how denial of pain medication deterred other patients from seeking the procedure: “…*many patients are opting to leave the ward minus the procedure when they discover how painful it is”* [79]. Poor attitudes and behaviours of health facility staff are also an important factor governing choice of facility at which to seek care [39, 42, 60, 66]. In a Nigerian survey, a substantial proportion of women who had recently delivered reported that poor staff attitudes, described as being unfriendly, disrespectful, and verbally abusive, were a reason for not using both ANC and delivery services offered at primary health care centres [67]. In Cambodia, Ghana, and South Africa, women and their families opted to seek care at private facilities even though services were costlier, or at facilities that were further away [60], because providers were known to be friendly and caring there [42, 60, 66].

Overall, negative attitudes and behaviours also have an impact on provision of health care. Unavailability or absenteeism of MHCPs are clearly barriers to health service access, specifically cited in two studies [67, 90]. Also, in a qualitative study in the Democratic Republic of Congo and a mixed qualitative-quantitative study in Tanzania, neglect by providers was reported to result in delays in receiving care once at the hospital [85, 100].

### Impact on maternal health outcomes

In seven studies, providers’ neglect or refusal to administer treatment was linked to increased risk of morbidity and mortality of women and their babies around the time of labour [61, 66, 71, 74, 79, 100, 101]. A study in Mozambique described how one participant had been refused delivery care whilst in labour “*and gave birth to a son on the roadside as she attempted to go back home. With her placenta still inside her and bleeding heavily, she had returned to the MC [maternity clinic]*” [101]. A case–control study exploring the circumstances of survivors and non-survivors of obstetric complications found that a higher percentage of survivors had received timely and appropriate care (40 % within 2 h and 85 % within 24 h) compared to non-survivors (19 % within 2 h and 44 % within 24 h) [100]. In another instance, neglect by doctors apparently led to the death of a patient, as narrated by a midwife in Gambia: “*She was brought to the hospital on the 13th at around 9:00 am from another health centre. The doctor saw her and diagnosed hand-presentation. He [doctor] asked us [midwives] to observe her. No action was taken by the doctors up to the 15th late in the evening [48 h later] when they took her to the theatre. He [doctor] first tried external cephalic version, which failed before a caesarean section was performed. The patient was wheeled dead from the theatre*” [71].

## Discussion

Although MHCP attitudes and behaviours have a considerable influence on women’s and their families’ perceptions of quality of care and thereby decisions to seek care, and ability to access appropriate and adequate maternal health care, surprisingly few studies have comprehensively sought to understand these issues in LMICs. The lack of interventional research on this topic is especially remarkable: no studies specifically aiming to alter MHCP attitudes or behaviours were identified.

Evidence synthesized from public and private health facilities in 42 LMICs across four regions (Africa, Asia, Latin America, Middle East) show frequent reporting of negative attitudes and behaviours, most commonly verbal abuse, rude behaviours and neglect. These were ascribed to a range of trained professionals, including doctors, nurses, midwives and paramedics, but reported predominantly in public rather than privately owned health facilities. The types of attitudes and behaviours did not vary significantly based on the stage of maternity care, with the exception of impatience and a lack of willingness to accommodate traditional practices which were reported only during delivery. These findings mirror those of a study included in this review which measured the frequency of reported abusive MHCP behaviours at the time of childbirth: 14 % of women sampled (n = 593) were ignored, 13 % verbally abused, and 12 % received negative and threatening comments [81]. Similarly, a comprehensive USAID-supported review of disrespect and abuse during childbirth in facilities, involving a review of published and grey literature as well as primary qualitative data collection, also noted instances of physical abuse, non-consented and non-confidential care, non-dignified care, discrimination based on specific patient attributes, abandonment of care, and detention of patients in facilities in LMICs [21]. Grey literature reports based on primary data collection from South Africa, Nigeria, Kenya and Peru, framed within the context of human rights, describe instances of neglect and refusal to provide care, verbal and physical abuse, as well as discrimination of women by MHCPs [110–114].

Positive attitudes and behaviours on the other hand, described as being caring, respectful, friendly, informative and sympathetic, were much less frequently reported. Evidence of such interactions was noted in Africa, Asia, Latin America and the Middle East, and primarily during ANC and at the time of childbirth, with no specific patterns observed in terms of type of facility or cadre of health worker.

As in the case of health worker performance and motivation [25, 26], this review found that MHCP attitudes and behaviours are complex phenomena, shaped by several macro- and micro-level interrelated factors: the broader cultural context, work conditions and the workplace environment, provider beliefs and characteristics, clients’ attitudes and behaviours, and the overall provider-client relationship (Fig. 1). Providers were more likely to be caring and understanding when they had a pre-existing relationship with the patient, or were familiar with the patient’s culture or community. Negative attitudes and behaviours often related to poor working conditions, which include heavy workloads, long working hours, and shortages of equipment and medicines (Fig. 1). Other key factors influencing negative attitudes were the provider attributes, beliefs and prejudices, as well as their perceptions of negative patient attitudes and behaviours, such as delayed care seeking or apparent lack of compliance with medical advice. Bowser and Hill reached similar conclusions in the USAID review, reporting that factors such as provider prejudice, demoralization related to poor working conditions, and provider status contributed to disrespect and abuse of women in facilities [21].

The most commonly reported impact of MHCP attitudes and behaviours was on care seeking. Women were more likely to attend ANC and deliver in a health facility when MHCPs had positive attitudes and behaviours. Conversely, when providers were rude and known to abuse patients, women were fearful and distressed, less satisfied with care, and likely to opt for home delivery with a traditional birth attendant. The latter are frequently described as helpful, caring and sympathetic [51, 74, 87, 88, 102]. Results of the few studies that provided quantitative data related to MHCP attitudes and behaviours support the qualitative evidence. Reluctance to attend ANC, delivery and postnatal care increases the risk of poor maternal and newborn health outcomes [115]. Also, fraught communication and relations between patients and providers will likely undermine the transfer of important maternal and newborn health promotion messages.

Of note, MHCPs’ neglect or refusal of care led to delays in appropriate and adequate care, which in turn increased risk of morbidity and mortality. A study in The Democratic Republic of Congo showed that women who died from obstetric complications were less likely to have received timely and appropriate care than women who survived [100]. It is also noteworthy that studies from developed and developing countries show that feeling a lack of control and support during labour can result in postpartum depression and post-traumatic stress disorder [116–118].

The effects of negative attitudes and behaviours on the promotion and protection of fundamental human rights, client satisfaction with care, and health outcomes highlight the need for program planning and service design. Such initiatives should take into account the complex factors which influence MHCP attitudes and behaviours. Many of these, such as cultural norms and provider and patient beliefs, will require context specific strategies. Others, such as inadequately equipped facilities or low provider salaries, will need to be addressed through overall health systems strengthening – particularly in relation to public health facilities. These efforts might include a review of human resource planning, provider roles and responsibilities, and financial incentives to determine how to minimize work-related stress for MHCPs.

Importantly, a rights-based approach must be consistently adopted when designing and delivering maternal healthcare. WHO defines such an approach as one where human rights norms and principles are included in the design, implementation, monitoring and evaluation of programmes and policy [119]. These norms and principles include human dignity, addressing the needs and rights of vulnerable groups, accessibility to health systems, and freedom from discrimination based on sex and gender roles [119]. This review however found several instances of pregnant women being disrespected and ignored, discriminated as a result of social norms and values, and denied access to health services. To uphold human rights in service design and implementation will again require addressing the factors which lead MHCPs to deny pregnant women and mothers their basic, fundamental rights.

Studies in this review highlighted that patients seek positive reinforcements, in the form of sympathy, care and understanding from health care providers, which help to promote care seeking [37–39, 43, 47–49, 54, 55]. Approaches, however, to promote positive MHCP attitudes and behaviours are presently under-developed, with evidence on the approaches tested to date available mainly from grey literature [21]. A few studies have noted improvements in provider self-esteem and provider-patient interactions following training for MHCPs on interpersonal and communication skills and patient engagement in childbirth [21, 120–123]. A WHO manual entitled ‘Counselling for maternal and newborn health care: a handbook for building skills’, is an example of a tool that might enhance provider communication skills [124]. Studies in Iran and Nepal also found that implementation of a strategy to improve process and structural elements of quality of care resulted in improved attitudes and behaviours [21, 125]. Other effective interventions might include: promoting supportive supervision of MHCPs by facility managers; professional development planning for MHCPs; ensuring accountability to professional standards and ethics at all levels of the health system; improving patients’ understanding of medical practices and their rights; and raising providers’ knowledge of local cultural practices in relation to pregnancy and childbirth [18]. Also potentially useful are international and national policies and advocacy around unacceptable provider behaviours, with a focus on human-rights based maternal health care [20, 126, 127].

Whilst the evidence on approaches related to maternity care may be limited, lessons learned in other areas, such as HIV/AIDS, may help to inform strategies to improve MHCP attitudes and behaviours. Studies in Nigeria, India, and Vietnam for example, found that training on changing knowledge and attitudes about HIV/AIDS [128, 129], and participatory processes whereby hospital staff develop action plans or policies to address stigma and discrimination [129, 130] helped to improve attitudes towards HIV positive patients. Similarly, in Uganda, an education program on HIV for nurses and nurse-midwives had a positive effect on professional practice, communication, and self-confidence [131].

### Limitations

This review is limited by inclusion of only English publications. The full-texts of eleven abstracts were also not available. Importantly, the study did not assess the quality of evidence, an important step in collating evidence with variable degrees of robustness. Assessment of quality of evidence was not done due to constrains in study resources and the complexity of assessing and comparing the quality of research across the different study designs included in the review. In addition, the higher numbers of reports of negative attitudes and behaviours than positive ones might be partly due to research generally focusing on system weaknesses, rather than on strengths. Also, the lack of quantitative studies limits our ability to quantify the impact of the attitudes and behaviours identified, particularly on maternal health outcomes. We were unable to examine differences in findings based on level of facility and other contextual factors affecting health worker attitudes and behaviours, such as those related to workload or the workplace environment, as the majority of studies did not provide these details. Many studies included here simply referred to health facilities or health workers in general, without specifying the type – thereby limiting the scope of the review’s findings. Lastly, the majority of relevant studies in this review were set in sub-Saharan Africa. While many MHCPs’ attitudes and behaviours may be common to other settings, differences in cultural and societal contexts may mean that effectiveness of potential interventions may vary across settings.

### Moving forward

Some gaps in evidence can be highlighted. More investigation is needed to better understand MHCP attitudes and behaviours in varied settings, factors promoting positive attitudes and behaviours, and the effectiveness of interventions to address negative patient experiences. More generally, maternal health system interventional research needs to include enquiry about potential impacts on MHCP attitudes and behaviours. Notable also is the predominance of studies in sub-Saharan Africa (around two thirds of all studies), highlighting the need for studies in other regions – particularly given that strategies to improve attitudes and behaviours will need to take into account contextual factors.

Findings of this review have important implications for the achievement of both MDG 4 and 5 and beyond, and suggest a need for markedly increased attention to this issue. Negative attitudes and behaviours constitute key deterrents to care seeking, as important as cost of services or geographical barriers. Disrespectful and abusive treatment of women also undermines ongoing efforts to increase skilled birth attendance [17]. The human rights violations resulting from such behaviour, namely the right to care, to health information, and freedom from physical abuse and neglect, equally demand a policy response [126, 127]. Positive attitudes and behaviours among MHCPs will not only contribute to improved maternal health outcomes, but may also help to reduce neonatal mortality and morbidity as a result of increased seeking of skilled care by pregnant women and mothers. Addressing provider attitudes and behaviours is therefore critical to ensuring continued progress towards the MDGs and saving the lives of women and children in low- and middle-income countries.

## Additional files

Additional file 1: **Supplementary material: search strategies.**

Additional file 2: Table S1. Characteristics and findings of included studies.

## Footnotes

1 A 15-item questionnaire which aims to identify patient experiences and problems with specific health care processes that affect the quality of care in inpatient settings (see Jenkinsen, Coulter & Bruster, 2002).
