# Supplementary material for: Attitudes and behaviours of maternal health care providers in interactions with clients: a systematic review
Source: Global Health. 2015 Aug 15;11:36. doi: 10.1186/s12992-015-0117-9 (PMC4537564; doi:10.1186/s12992-015-0117-9)
Supplement: Supplementary file 1 — Supplementary material: search strategies. [file 12992_2015_117_MOESM1_ESM.docx]

# SUPPLEMENTARY TABLE 1: CHARACTERISTICS AND FINDINGS OF INCLUDED STUDIES

**Abbreviations:** TBA=traditional birth attendant, ANC=antenatal care, NGO=non-governmental organization, C = client perspective, P = provider perspective, S = based on study observations, ST = statistical analysis, ANC = antenatal care, D = delivery, PNC = postnatal care, FP = family planning, AB = abortion, NS = not specified

| **#**  **(Ref #)** | **First author & Publication year** | **Setting** | **Study Design** | **Study Sample** | **Positive/ Negative attitude behaviour** | **Facility type & health worker cadre** | **Type of attitude or behaviour (stage: ANC, D, PNC, FP, AB, NS)** | **Influence(s) on attitudes and behaviours**  **(C/P/S/ST)** | **Impact(s) of attitudes and behaviours** |
| --- | --- | --- | --- | --- | --- | --- | --- | --- | --- |
| **1**  **(1)** | Adams et al 2005 | China | Qualitative: Semi-structured interviews | 38 village women, aged 18 – 40 years | Negative | Public hospitals  Health care providers | Rude (D) | Perceptions of patient ignorance (S)  Socioeconomic status of client (S)  Education level of client (S) | Patients offended  Fear of health care providers  Barrier to facility based delivery |
| **2**  **(2)** | Afsana et al 2001 | Bangladesh | Qualitative:  IDIs  FGDs  Observations | 15 women who had delivered over last 2 years, aged 20 – 40 years;  4 doctors;  7 female paramedics/nurses | Negative | Public health facilities  Private health facilities  Nurses  Health care providers | Poor communication (D)  Unwilling to accommodate traditional practices (D)  Lack of regard for privacy (D)  Verbal abuse (D)  Rude (D) | Lack of understanding of cultural practices (S)  Lack of training (S)  Socioeconomic status of client (S)  Workload (S) | Women feeling as passive objects  Fear of health care providers  Barrier to facility based delivery |
|  |  |  |  |  | Positive | Private health facilities  Health care providers | Caring (D)  Supportive (D) | Familiarity with community (S) |  |
| **3**  **(3)** | Agus et al 2012 | Indonesia | Qualitative:  FGDs | 16 women currently pregnant or had given birth | Negative | Home delivery  Midwives | Impatient, rushing childbirth (D) |  | Preference for TBAs over midwives |
| **4**  **(4)** | Akin-Otiko et al 2011 | Nigeria | Qualitative:  FGDs | 65 married women aged 15 – 49 years | Negative | Public facilities: Primary health care centers  Rural hospitals  Midwives | Rude (ANC, D)  Neglect (ANC)  Verbal abuse (D, ANC)  Seeking bribes (ANC, PNC) | Marital status of provider (C)  Age of provider (C)  Workload (C)  Workplace environment (C) |  |
|  |  |  |  |  | Positive |  | Encouraging (ANC)  Caring (ANC)  Cheerful (ANC)  Polite (ANC) |  |  |
| **5**  **(5)** | Amooti-Kaguna et al  2000 | Uganda | Qualitative:  FGDs  Semi-structured interviews | 275 participants:  32 men 243 mothers, of whom 211 were aged 15 – 45 years | Negative | Public health facilities  Nurses  Midwives | Neglect (D)  Rude (D)  Verbal abuse (D) |  | Barrier to facility based delivery |
|  |  |  |  |  | Positive | Private health clinics  Midwives  Doctors | Caring (D) |  |  |
| **6**  **(6)** | Asuquo et al  2000 | Nigeria | Qualitative:  FGDs | Women of childbearing age;  Men;  Health care providers | Negative | Private (university) hospital  Doctors  Nurses | Verbal abuse (D)  Discrimination (D) | Patient known to health worker (C)  Late health care seeking of patients (P)  Low job satisfaction (P)  Workplace environment (P)  Insufficient salaries (P) | Barrier to seeking obstetric care at the hospital |
| **7**  **(7)** | Atuyambe et al 2009 | Uganda | Qualitative:  FGDs  KIIs | 92 adolescent girls aged 16-19 years who were pregnant for 1^st^ time or delivered for first time over last 6 months | Negative | Public health facilities  Health care providers | Rude (ANC, D)  Verbal abuse (D)  Neglect (ANC)  Physical abuse (D) |  | Barrier to seeking ANC and facility based delivery |
|  |  |  |  |  | Positive |  | Sympathetic (D) |  | Seeking of health care |
| **8**  **(8)** | Avortri et al 2011 | Ghana | Survey | 885 women aged 18+ years who delivered vaginally | Negative | Public hospitals  Hospital staff | Rude (D) |  | Women treated with respect sometimes were 3.6 times more likely to be dissatisfied with childbirth care than those always treated with respect |
| **9**  **(9)** | Boller et al 2003 | Tanzania | Survey | 166 women attending public facilities  188 women attending private facilities | Positive | First-level public and private ANC services  Health care providers | Respectful (ANC)  Welcoming (ANC)  Polite (ANC)  Informative (ANC) |  | Of women attending public facilities (n=166), 93% were shown interest by providers, 70% were not interrupted, 98% felt providers were polite, and 71% were asked about their concerns  For private facilities (n=188), same figures were 95%, 87%, 98%, and 81% respectively |
| **10**  **(10)** | Brookes 1991 | South Africa | Qualitative:  KIIs | 15 women aged 16 – 35 years who delivered over last 7 – 14 days | Negative | Public provincial hospital and private hospital  Midwives | Verbal abuse (D)  Authoritarian (D)  Neglect (D) |  | Fear of health care providers |
| **11**  **(11)** | Campero et al 1998 | Mexico | Qualitative:  IDIs | 16 women who had delivered in the last 6 weeks | Negative | Public hospitals  Doctors  Nurses  Hospital staff | Authoritarian (D)  Frightening (D) |  |  |
| **12**  **(12)** | Chadwick et al 2014 | South Africa | Qualitative: interviews | 33 low-income women aged 18–42 years who had recently given birth | Negative | Public health facilities  Nurses  Midwives | Verbal abuse (D)  Rude (D)  Neglect (D)  Lack of communication (D) |  | Fear of health care providers  Distress among patients  Patients feeling shamed and humiliated |
| **13**  **(13)** | Cham et al 2005 | Gambia | Qualitative:  Deaths review  Verbal autopsy | 42 cases of maternal deaths, verbal autopsy performed on 32 of the deaths  Interviews undertaken with relatives of the deceased and health workers | Negative | Type of facility not specified  Nurses | Verbal abuse (ANC) |  | Delay in seeking facility based care |
| **14**  **(14)** | Chapman 2003 | Mozambique | Qualitative (KIIs) and quantitative (survey) | 83 women aged 15-49 years who were pregnant or who had given birth | Negative | Public maternity clinics  Clinic staff | Refusal to provide care (ANC, D) |  | Barrier to seeking facility based care  Seeking care only to obtain ANC card  Increased risk of morbidity or mortality |
| **15**  **(15)** | D’Ambruoso et al  2005 | Ghana | Qualitative: IDIs  FGDs | Women between 18 and 36 years who had delivered over last 5 years with a skilled professional | Negative | Public and private health facilities  Midwives  Nurses  Birth attendant  Facility staff | Rude (D)  Impatient (D)  Physical abuse (D) |  | Seeking ANC to obtain ANC card  Influence on choice of facility for delivery  Impact on client satisfaction |
|  |  |  |  |  | Positive |  | Caring (D)  Helpful (D)  Sympathetic (D) |  |  |
| **16**  **(16)** | Duong et al 2004 | Viet Nam | Mixed qualitative (FGDs and IDIs) and quantitative (survey) | 200 women who had delivered in the previous 3 months (quantitative)  Women who had delivered in the previous 3 months, mothers/mothers-in-law, husbands/ partners, public and private providers, TBAs, women’s union activists (qualitative) | Negative | Public community health centres and district hospitals  Health care workers | Verbal abuse (NS)  Rude (NS) |  | Barrier to seeking facility based care |
| **17**  **(17)** | Foster et al 2010 | Dominican Republic | Qualitative: FGDs  KIIs | 51 adolescent females 15-20 years who had given birth at public hospitals;  59 adult females 21–49 years who had given birth at a public hospital;  27 males aged 19+ years | Negative | Public hospitals  Doctors  Nurses  Medical staff | Neglect (ANC, D)  Rude (D)  Lack of communication (D)  Discrimination (ANC, D)  Seeking bribes (NS) | Patient known to health worker (C) |  |
| **18**  **(18)** | Gao et al 2010 | China | Qualitative:  Semi-structured interviews  Observations | 30 women who had given birth in the previous six months | Negative | Public health facilities  Doctors  Nurses  Midwives | Neglect (D)  Impatient, rushing childbirth (D)  Seeking bribes (D) |  | Barrier to facility based delivery |
| **19**  **(19)** | Gebrehiwot et al  2012 | Ethiopia | Qualitative : FGDs | 51 women aged 15 to 40 years who had given birth in the previous 3 years | Negative | Public health facilities  Health workers | Verbal abuse (D)  Rude (D, PNC) |  | Barrier to facility based delivery |
| **20**  **(20)** | Gilson et al 1994 | Tanzania | Qualitative:  KIIS  IDIs  FGDs  Observations | Mothers;  Village chairmen; Traditional healers;.  TBAs;  Chairladies of women’s organizations; Respected elders; Religious leaders; Chronically ill persons;  Poor persons | Negative | Public dispensaries  Nurses  Health care providers | Neglect (ANC)  Rude (ANC, FP)  Discrimination (ANC) |  |  |
|  |  |  |  |  | Positive | Private (church) dispensaries  Health care providers | Polite (ANC, D)  Respectful (ANC, D) |  |  |
| **21**  **(21)** | Grossman-Kendall et al 2001 | Benin | Qualitative: IDIs | 19 women aged 20 – 40 years who had delivered in the last 5 – 9 months | Negative | Public & private (university) hospitals  Midwives  Health personnel | Rude ANC, D)  Lack of communication (ANC, D)  Physical abuse (D) | Age of provider (C)  Age of client (P)  Traditional norms (S)  Perceptions of pregnancy and delivery (S) | Barrier to seeking ANC  Poor quality interactions and absence of trust between patients and midwives |
|  |  |  |  |  | Positive | Midwives | Caring (D) |  | Patient satisfaction with care |
| **22**  **(22)** | Hadwiger et al 2012 | Philippines | Qualitative : interveiws | 26 mothers who had used TBAs for childbirth | Negative | Public hospitals  Doctors  Nurses | Rude (D)  Verbal abuse (D)  Lack of privacy (D)  Authoritarian (D) | Workload (C) | Barrier to facility based delivery  Preference for home births |
| **23**  **(23)** | Hassan-Bitar et al 2011 | Palestine | Mixed qualitative (IDIs, observations) and quantitative | 9 midwives;  14 nurses;  8 doctors | Negative | Public hospital  Nurses  Midwives | Lack of communication (D)  Rude (D) | Workload (P)  Education level of provider (P) |  |
| **24**  **(24)** | Igboanugo et al 2011 | Nigeria | Qualitative: KIIs | 8 pregnant women aged 24 – 35 years | Negative | Public health facilities  Nurses  Doctors | Verbal abuse (D) |  | Barrier to facility based delivery |
| **25**  **(25)** | Ith et al  2013 | Cambodia | Qualitative: IDIs | 30 women who had recently given birth | Negative | Public & private facilities  Midwives  Nurses | Neglect (D)  Rude (D)  Verbal abuse (D)  Lack of communication (D)  Lack of privacy (D)  Physical abuse (D) | Socio-economic status of client (C) | Barrier to facility based delivery  Preference for private care  Seeking care at costlier facilities further away |
| **26**  **(26)** | Izugbara et al 2009 | Kenya | Qualitative: FGDs | 23 self-identified TBAs aged 28 to 70 years | Negative | Public hospitals  Doctors  Health facility staff | Seeking bribes (D)  Neglect (D)  Verbal abuse (D)  Rude (D)  Lack of regard for traditional practices (D)  Lack of consideration of patient’s socio-economic status (D) |  | Barrier to facility based delivery  Increased risk of morbidity and mortality |
| **27**  **(27)** | Jaffre et al 1994 | Niger | Qualitative: FGDs | 60 women  Experienced midwives; Student midwives | Negative | Public health facilities  Midwives  Nurses | Neglect (D)  Verbal abuse (D)  Physical abuse (D) | Cultural norms (P)  Patient attitudes and behaviours (P) |  |
| **28**  **(28)** | Jewkes et al 1998 | South Africa | Qualitative: IDIs  FGDs | Pregnant women;  9 midwives;  3 nurses;  1 family planning worker;  Health care providers | Negative | Public health facilities  Midwives  Nurses  Health care providers | Verbal abuse (ANC< D)  Physical abuse (D)  Neglect (D)  Discrimination (D) | Relations with co-workers (P)  Patient attitudes and behaviours (P)  Power assertion (P)  Clinical practice (P)  Perceptions of patient ignorance (P)  Provider-patient hierarchies (P)  Workplace environment (P)  Workload (P)  Low job satisfaction (P) | Preference for private care  Fear and distress among patients |
|  |  |  |  |  | Positive | Midwives | Caring (D)  Supportive (D)  Friendly (D) |  |  |
| **29**  **(29)** | Kaartinen et al 2002 | Afghanistan | Mixed qualitative (semi-structured interviews) and quantitative (survey) | 100 women who had delivered over last 10 years, aged 15 – 26 years | Negative | Public health facilities  Doctors  Nurses  Health care providers | Poor communication (NS)  Rude (D)  Neglect (D)  Seeking bribes (D) | Lack of supplies (S) |  |
| **30**  **(30)** | Kabakian-Khasholian et al 2000 | Lebanon | Qualitative: Semi-structured interviews | 117 women aged 19 – 40 years who had delivered in the last three months | Negative | Public & private facilities  Nurse  Doctor | Lack of communication (D)  Neglect (D)  Verbal abuse (D)  Impatient, rushing childbirth (D)  Lack of privacy (D) |  |  |
|  |  |  |  |  | Positive | Public & private facilities  Obstetrician  Midwife  Doctor | Caring (ANC, D)  Informative (ANC)  Supportive (D)  Encouraging (D) |  | Satisfaction with care  Higher patient self esteem |
| **31**  **(31)** | Kabali et al 2011 | Democratic Republic of Congo | Qualitative: semi-structured interviews | 211 maternal deaths;  358 controls | Negative | Public hospitals  Doctors  Nurses | Neglect (D)  Seeking bribes (D) |  | Delays in obtaining care once at the hospital  Increased risk of morbidity or mortality |
| **32**  **(32)** | Kempe et al 2010 | Yemen | Survey | 220 women with childbirth experience | Negative | Type of facility not specified  TBA;  Primary Health Care Facilitators; Midwives;  Registered Nurse Midwives;  Doctors | Rude (D) | Level of clinical training (ST) | Patients have less authority over childbirth |
| **33**  **(33)** | Kowalewski et al 2000 | Tanzania | Qualitative: semi-structured interviews | Sixty pregnant women;  26 Health care providers;  3 senior health officials;  2 traditional midwives;  1 teaching staff | Negative | Public health facilities  Health care providers | Neglect (D)  Discrimination (D)  Impatient, rushing childbirth (D) |  | Barrier to seeking facility based care |
| **34**  **(34)** | Kruk et al 2014 | Tanzania | Survey | 1,779 women aged 15 years and older who had delivered at the facilities sampled at discharge,  593 women who had delivered at the facilities 8 weeks following discharge | Negative | Public and private district hospitals, public health centres and one public dispensary | Verbal abuse (D)  Neglect (D)  Lack of regard for privacy (D)  Rude (D)  Physical abuse (D)  Seeking bribes (D) | Education level of client (ST)  Marital status of client (ST)  Client history of physically abuse (ST)  Client mood in the previous 12 months (ST)  Socioeconomic status of client (ST)  Delivery status (complications, caesarean section) (ST)  Patient is primiparous (ST)  Duration of stay in the facility (ST) |  |
| **35**  **(35)** | Kumbani et al 2013 | Malawi | Qualitative: IDIs | 12 women aged 20 to 32 years who had delivered outside a health facility | Negative | Health centre (public or private unknown)  Doctors  Health workers | Verbal abuse (ANC, PNC)  Rude (ANC, PNC, D)  Neglect (D) | Patient attitudes and behaviours (C) | Barrier to seeking facility based care  Preference for delivery with TBAs |
|  |  |  |  |  | Positive |  | Respectful (ANC)  Welcoming (ANC)  Attentive (PNC) |  | Seeking facility based ANC |
| **36**  **(36)** | Kyomuhendo et al 2003 | Uganda | Mixed qualitative (FGDs, KIIs) and quantitative (survey) | 808 women;  240 men and women;  20 elders  Health workers (# not specified) | Negative | Public facilities: primary health units, referral hospitals  Doctors  Midwives  Health care providers | Unwilling to accommodate traditional practices (D)  Impatient, rushing childbirth (D)  Rude (D)  Seeking bribes (D) | Patient attitudes and behaviours (P)  Clinical training (P) | Barrier to facility based delivery |
| **37**  **(37)** | Langer et al 1998 | Cuba, Thailand, Argentina | Qualitative: FGDs,  IDIs | 212 pregnant women | Negative | Public health facilities  Doctors | Verbal abuse (ANC) |  |  |
| **38**  **(38)** | Larsen et al 2004 | Papua New Guinea | Qualitative: Semi-structured interviews | 20 pregnant women | Negative | Public health facilities  Health care providers | Rude (ANC) | Lack of space at the facility (P) |  |
| **39**  **(39)** | Lubbock et al  2008 | Nicaragua | Qualitative:  IDIs | 37 non-pregnant women who had children under the age of 3, aged 18 – 40 years | Negative | Type of facility not specified  Health care providers | Neglect (ANC, D) |  | Barrier to facility based delivery |
| **40**  **(40)** | Mackeith et al 2003 | Zambia | Survey | 1,210 women | Negative | Type of facility not specified  Midwives  Doctors | Neglect (D)  Verbal abuse (D) |  |  |
|  |  |  |  |  | Positive |  | Kind (D)  Supportive (D)  Understanding (D) |  |  |
| **41**  **(41)** | Magoma et al 2010 | Tanzania | Qualitative:  KIIs  FGDs  Participant observation | 18 care providers (15 of which trained to the level of nurse midwife); 36 TBAs; 40 male elders; 66 women who had sought ANC, delivery, and PNC services | Negative | Two private district hospitals, seven health dispensaries  Health care providers | Verbal abuse (D)  Lack of communication (D)  Physical abuse (D) |  | Preference for home births with TBAs |
|  |  |  |  |  | Positive |  | Caring (D) |  | Seeking facility based delivery |
| **42**  **(42)** | Mathole et al 2004 | Zimbabwe | Qualitative: FGDs  Interviews | 68 women, aged 19–46 years, and men aged 24 - 52 years | Negative | Public health facilities  Nurses | Verbal abuse (D)  Rude (D) |  | Barrier to facility based delivery  Barrier to accepting hospital referral  Delays in care seeking |
| **43**  **(43)** | McMahon et al 2014 | Tanzania | Qualitative: IDIs | 49 women with uncomplicated deliveries in the preceding 14 months, 27 male partners, 20 community health workers, 5 community leaders, 11 religious leaders | Negative | Public health facilities  Health care providers | Neglect (D)  Verbal abuse (D)  Seeking bribes (D)  Physical abuse (D) | Workload (C) | Barrier to facility based delivery  Preference for home delivery |
| **44**  **(44)** | Medeiros et al 2013 | Brazil | Qualitative: IDIs | 5 women who had undergone abortion | Negative | Public hospital  Doctors | Lack of communication (AB) |  |  |
|  |  |  |  |  | Positive | Public hospital  Doctors  Health staff | Caring (AB)  Friendly (AB) |  |  |
| **45**  **(45)** | Miller et al 2003 | Dominican Republic | Mixed qualitative (KIIs, FGDs, observations) and quantitative (audits, survey) | 57 prenatal patients;  55 women in labor;  21 women having vaginal deliveries;  6 women having caesarean deliveries;  88 maternity care providers | Negative | Public health referral level facilities  Doctors  Nurses  Facility staff | Neglect (D)  Lack of communication (D)  Lack of privacy (D)  Verbal abuse (D)  Physical abuse (D)  Impatient, rushing childbirth (D) | Workload (P)  Patient attitudes and behaviours (P) |  |
| **46**  **(46)** | Moore et al 2002 | Kenya | Qualitative: IDI  FGDs  GDs | 14 women with normal delivery in last 6 months;  4 women who had obstetric complications; 6 elder female family influentials;  6 Husbands or male partners;  9 TBAs;  10 Skilled birth attendants;  Community leaders | Negative | Public health facilities  Doctors  Nurses  Health care providers | Verbal abuse (ANC, D)  Physical abuse (ANC, D)  Neglect (D)  Discrimination based on socio-economic status (ANC, D) | Insufficient salaries (P) | Barrier to facility based delivery  Increased risk of mortality |
| **47**  **(47)** | Mrisho et al 2007 | Tanzania | Mixed qualitative (IDIs, FGDs, observations) and quantitative (survey) | Women who had recently delivered at home or at a health facility¨  9,152 women for survey  32 FGDs with 6 – 8 women | Negative | Public health facilities  Midwives  Nurses  Health care providers | Verbal abuse (D)  Neglect (D)  Refusal to provide care (D)  Rude (D) |  | Seeking ANC to obtain ANC card  Barrier to facility based delivery |
|  |  |  |  |  | Positive | Public health facilities  Midwives | Polite (D)  Sympathetic (D) |  | Seeking facility-based delivery |
| **48**  **(48)** | Mselle et al 2011 | Tanzania | Mixed qualitative (interviews) and quantitative (survey) | 16 women affected by obstetric fistula;  151 women admitted in the fistula wards | Negative | Health facilities  Nurses | Rude (D)  Neglect (D) |  | Delay or barrier to facility based delivery  Delay in receiving care once at a facility |
| **49**  **(49)** | Mumtaz et al 2003 | Pakistan | Qualitative (IDIs) | 8 lady health workers; ;  5 TBAs;  8 lady health visitors ;  4 nurses;  5 doctors | Negative | Public health system  Lady health workers | Rude (ANC, FP)  Seeking bribes (ANC, FP) | Gender norms (P)  Insufficient salaries (P)  Low motivation (P) |  |
| **50**  **(50)** | Murira et al 2003 | Zimbabwe | Qualitative: interviews  observations | 10 pregnant women aged 14 to 20 referred to hospital for complications, doctors, midwives, health staff in contact with pregnant women | Negative | Antenatal clinic in a referral level public hospital  Doctors  Midwives | Lack of communication (ANC)  Lack of privacy (ANC)  Authoritarian (ANC) | Provider-patient hierarchies (C, S)  Patient age (C)  Workload (C) | Fear and distress |
| **51**  **(51)** | Mwangome et al 2012 | Kenya | Qualitative:  Structured interviews  GDs | 90 community members aged 20 – 60 years;  12 hospital staff;  26 mothers who opted not to deliver in hospitals | Negative | Public hospital  Nurses | Neglect (D)  Verbal abuse (D)  Physical abuse (D) |  |  |
| **52**  **(52)** | Nabukera 2006 | Uganda | Mixed qualitative (FGDs, IDIs) and quantitative (survey) | 31 health care providers;  32 TBAs;  112 mothers who delivered in the last three years | Negative | Public health facilities  Health care providers | Neglect (D)  Rude (D) |  | Barrier to facility based delivery |
| **53**  **(53)** | Ngomane et al 2012 | South Africa | Qualitative: IDIs | 12 pregnant women | Negative | Public hospitals, health facilities  Nurses | Verbal abuse (ANC, D)  Discrimination (ANC)  Rude (ANC, D) |  | Barrier to facility based delivery |
| **54**  **(54)** | Nguyen et al 2007 | Viet Nam | Mixed qualitative (IDIs, interviews, observations)and quantitative (survey) | 748 women undergoing an abortion;  7 health care staff | Negative | Public health facilities  Doctors  Facility staff | Rude (AB)  Verbal abuse (AB)  Neglect (AB)  Lack of communication (AB)  Lack of privacy (AB) | Patient-provider hierarchies (P)  Lack of space at health facility (P)  Workplace environment (P)  Lack of training (P)  Lack of supplies (P)  Patient attitudes and behaviours (P) |  |
| **55**  **(55)** | Nigenda et al 2003 | Argentina  Cuba  Saudi Arabia  Thailand | Qualitative: FGDs | 164 pregnant women who completed 2 ANC visits | Negative | Public health facilities  Doctors | Rude (ANC)  Seeking bribes (ANC) |  |  |
|  |  |  |  |  | Positive | Public health facilities  Doctors  Health staff | Respectful (ANC)  Polite (ANC)  Generous (ANC) |  |  |
| **56**  **(56)** | Okafor et al 1994 | Nigeria | Qualitative: FGDs  Interviews | Married women & men;  Adolescents; TBAs;  Midwives; Auxiliary midwives | Negative | Public health facilities  Nurses  Midwives | Verbal abuse (ANC, D)  Rude (ANC, D)  Refusal to provide care (ANC, D) | Workload (P)  Insufficient salaries (P)  Low motivation (P) | Barrier to facility based deliveries  Preference for home births |
| **57**  **(57)** | Onah et al 2006 | Nigeria | Survey | 1095 women who had delivered in the 3 preceding months | Positive | Public & private health facilities  Health care providers | Friendly (D) |  | Seeking facility-based delivery |
| **58**  **(58)** | Otis et al  2008 | Bolivia | Qualitative: participant observation, KIIs, semi-structured interviews | 44 mothers and 18 fathers of children under 5 years  8 regional experts in health care provision | Negative | Public hospital and health facilities  Doctors  Nurses | Verbal abuse (D)  Neglect (D) |  | Barrier to facility based deliveries |
| **59**  **(59)** | Oyerinde et al  2012 | Sierra Leone | Qualitative: FGDs | 96 women aged 15 to 60 years; 64 men aged 16 to 49 years | Negative | Public referral-level hospitals  Nurses  Health care providers | Verbal abuse (D)  Neglect (D)  Impatient, rushing childbirth (D) |  | Barrier to facility based deliveries  Preference for home births with TBAs |
|  |  |  |  |  | Positive |  | Caring (D) |  | Seeking facility-based delivery |
| **60**  **(60)** | Pell et al 2013 | Ghana  Kenya  Malawi | Qualitative:  FGDs  Interviews  Observations | 352 pregnant women;  105 health providers;  91 relatives;  46 opinion leaders;  51 community members | Negative | Public health facilities  Private hospitals  Health care providers | Verbal abuse (ANC)  Discrimination (ANC) | Socioeconomic status of client (S)  Education level of client (S)  Patient known to health worker (S) | Seeking ANC out of fear  Seeking ANC to obtain ANC card |
| **61**  **(61)** | Pettersson et al 2006 | Mozambique | Qualitative:  IDIs | 16 midwives aged 29 to 42 years | Negative | Public health facilities  Midwives | Rude (D)  Frightening (D)  Frustration (D) | Education level of client (P)  Socioeconomic status of client (P)  Patient attitudes and behaviours (P) |  |
| **62**  **(62)** | Pretorius et al 2004 | South Africa | Mixed quantitative (survey) and qualitative (semi-structured interviews) | 26 chief nurses working in antenatal services; pregnant women who had given birth over last 5 days | Positive | Public ANC facilities  Nurses | Caring (ANC) |  | Seeking ANC |
|  |  |  |  |  | Negative |  | Verbal abuse (ANC, FP)  Physical abuse (ANC, FP) |  | Barrier to seeking ANC and family planning services |
| **63**  **(63)** | Pryterch et al 2013 | Burkina Faso  Ghana  Tanzania | Qualitative: IDIs | 75 MNH providers;  24 facility and district managers;  6 policy-makers | Negative | Public & private health facilities  Doctors  Midwives  Auxiliary midwives  Outreach workers | Verbal abuse (D)  Physical abuse (NS)  Seeking bribes (NS) | Patient attitudes and behaviours (P)  Personal financial pressures of provider (P)  Provider-patient hierarchies (P)  Workload (P)  Age of provider (P) |  |
| **64**  **(64)** | Rahmani et al 2013 | Afghanistan | Qualitative: Semi-structured interviews | 12 pregnant women or women who had recently given birth;  7 doctors;  5 midwives;  3 TBAs | Negative | Public hospital  Private clinics  Doctors  Midwives  Health care providers | Verbal abuse (ANC, D)  Physical abuse (D)  Lack of communication (ANC)  Refusal to provide care (FP)  Discrimination (NS)  Seeking bribes (NS) | Patient – provider hierarchies (P)  Workload (P)  Insufficient salaries (P)  Workplace environment (P) | Fear of health care providers  Barrier to seeking reproductive services at public facilities |
| **65**  **(65)** | Rai et al 2011 | India | Qualitative: FGDs  IDIs | 144 mothers who delivered at a health facility;  144 who delivered at home;  12 village health and sanitation committee members;  Mother-in-laws;  Father-in-laws/husbands | Negative | Public health facilities  Doctors  Nurses  Health care providers | Seeking bribes (D)  Neglect (D)  Lack of communication (D) |  | Barrier to facility based delivery |
| **66**  **(66)** | Ruiz et al 2012 | Guatemala | Qualitative: Interviews | 18 users of MWH services;  5 influential family members;  4 community leaders;  5 MWH administrative and medical staff;  7 comadronas; 2 medical staff; 1 district-level ministry of health representative; 6 medical personnel | Negative | Public facilities: maternity waiting homes, referral hospital  Hospital staff | Verbal abuse (D)  Lack of regard for cultural practices (D) |  | Barrier to using maternity waiting homes |
| **67**  **(67)** | Seljeskog et al 2006 | Malawi | Qualitative: IDIs  Observations | 3 women who delivered at home, 3 at a hospital;  2 Health care providers;  2 TBAs | Negative | Public health facilities  Nurses | Neglect (D)  Lack of communication (D) | Workload (P)  Socioeconomic status of client (P, S)  Education level of client (P, S) | Seeking ANC to obtain ANC card |
| **68**  **(68)** | Shiferaw et al 2013 | Ethiopia | Mixed qualitative (IDIs & FGDs) and quantitative (survey) | 909 women (survey);  21 health care providers;  2 TBAs;  8 mothers who recently delivered;  8 partners of women who recently delivered | Negative | Public health facilities  Health care workers | Lack of privacy (D)  Lack of regard for cultural practices (D)  Rude(D)  Neglect (D) |  | Barrier to facility based delivery |
| **69**  **(69)** | Silal et al  2011 | South Africa | Mixed qualitative (IDIs) and quantitative (survey) | Quantitative:  632 women from urban and 599 women from rural sub-district obstetric facilities, above the age of 18 who had been discharged  Qualitative:  16 women who had recently given birth | Negative | Public health facilities  Health care workers | Verbal abuse (ANC, D)  Refusal to provide care (D)  Lack of communication (D)  Rude (PNC) | Workload (C)  Insufficient salaries (C) |  |
| **70**  **(70)** | Smith et al 2004 | Zambia | Qualitative: IDIs | 24 women with disabilities;  25 public reproductive health service providers | Negative | Public health facilities  Health facility staff | Rude (ANC, FP) | Lack of training (P) | Barrier to seeking facility based care |
| **71**  **(71)** | Solo 2000 | Kenya | Review | -13 studies | Negative | Type of facility not specified  Doctors | Physical abuse (AB)  Verbal abuse (AB) | Provider views on abortion (P) | Patient in pain |
| **72**  **(72)** | Spangler et al 2010 | Tanzania | Mixed qualitative (IDIs, participant observation) and quantitative (statistical analysis on secondary data) | Quantitative : 1150 women  Qualitative : 48 women who delivered within last 6 months (IDIs), all women 14 years and above with childbirth experience in 3 central villages in 2 districts | Negative | Two district hospitals, two village health centers, health dispensaries (public and private)  Health care workers | Verbal abuse (D)  Neglect (D)  Rude (D) | Patient socio-economic status (C)  Patient ethnicity (C)  Patient known to health worker (C) | Barrier to seeking facility based obstetric care |
| **73**  **(73)** | Tilahun et al | Ethiopia | Survey | 401 Health care providers | Negative | Public health facilities  Nurses  Doctors  Health assistants |  | Education level of provider (ST)  Marital status of provider (ST)  Lack of training (ST)  Use of family planning methods by provider (ST)  Patient attitude and behaviours (P) |  |
| **74**  **(74)** | Titaley et al  2010 | Indonesia | Qualitative: FGDs  IDIs | 119 mothers of children aged 40+ days – 4 months;  40 fathers;  26 health professionals | Negative | Home delivery  Midwives | Impatient (D)  Rude (D) | Age of provider (C)  Level of experience (C) | Preference to deliver with TBAs |
| **75**  **(75)** | Tlebere et al 2007 | South Africa | Mixed qualitative (semi-structured interviews, case studies) and quantitative (interviews) | 235 HIV positive and negative mothers who had delivered in the last 9 -12 months | Negative | Public health facilities  Nurses | Rude (ANC) |  | Impact on seeking ANC |
|  |  |  |  |  | Positive |  | Friendly (ANC)  Motivating (ANC) |  |  |
| **76**  **(76)** | Tuncalp et al 2012 | Ghana | Mixed qualitative (semi-structured interviews) and quantitative (survey) | 32 women who had experienced severe maternal morbidity | Negative | Referral level urban hospital  Doctors  Midwives  Health care workers | Neglect (D)  Rude (D) |  |  |
|  |  |  |  |  | Positive | Referral level urban hospital  Doctors | Informative (D)  Respectful (NS) |  |  |
| **77**  **(77)** | Uzochukwu et al 2004 | Nigeria | Mixed qualitative (FGDs) and quantitative (survey) | 405 women who had a child under 1 year old, or had a live delivery over the last year;  18 Men | Negative | Public health facilities  Nurses | Verbal abuse (ANC, D)  Rude (ANC, D) | Workload (C)  Encounters with sickness and death (C) | Poor satisfaction of care  Barrier to seeking facility based care |
| **78**  **(78)** | Vera 1993 | Chile | Qualitative: IDIs | 60 women receiving services at a maternal and child health clinic | Positive | NGO clinic  Clinic staff | Respectful (FP)  Courteous (FP) |  |  |
| **79**  **(79)** | Weeks et al 2005 | Uganda | Qualitative: Semi-structured interviews | 30 women who narrowly avoided maternal death | Negative | Public hospital  Health care providers | Verbal abuse (D)  Neglect (D)  Lack of communication (D) |  | Risk of mortality |
|  |  |  |  |  | Positive |  | Polite (D) |  |  |
| **80**  **(80)** | Wild et al | Timor Leste | Qualitative: interviews, group discussions | 21 pregnant women aged 18 to 43 years  14 mothers of babies  5 husbands  Families of the women: 2 mothers, 1 grandmother, 3 sisters, 2 mother-in-laws  Health facility and NGO managers (# not specified) | Negative | Public birthing facilities (hospitals, health centres)  Midwives | Rude (D)  Verbal abuse (D)  Neglect (D) | Patient is primiparous (P) | Barrier to facility based delivery |
|  |  |  |  |  | Positive |  | Caring (D) |  | Seeking of facility based care |
| **81**  **(81)** | Yakong et al 2010 | Ghana | Qualitative: IDIs  FGDs  Observations | 27 women aged 15 – 49 years | Negative | Public and private clinics  Nurses  Clinic staff | Verbal abuse (ANC, D)  Rude (ANC, D)  Lack of privacy (NS) |  | Barrier to seeking care  Preference for private care |

**References**

1. Adams, V., S. Miller, J. Chertow, S. Craig, A. Samen, M. Varner, Having a "safe delivery": conflicting views from Tibet. Health care for women international, 2005. 26(9): p. 821-51.

2. Afsana, K., S.F. Rashid, The challenges of meeting rural Bangladeshi women's needs in delivery care. Reproductive health matters, 2001. 9(18): p. 79-89.

3. Agus, Y., S. Horiuchi, S.E. Porter, Rural Indonesia women's traditional beliefs about antenatal care. BMC research notes, 2012. 5: p. 589.

4. Akin-Otiko, B.O., B.R. Bhengu, Client education experiences and expectations of women at the first level of maternal and child care in Kaduna state, Nigeria. Midwifery, 2012. 28(6): p. e893-9.

5. Amooti-Kaguna, B., F. Nuwaha, Factors influencing choice of delivery sites in Rakai district of Uganda. Social science & medicine, 2000. 50(2): p. 203-13.

6. Asuquo, E.E.J., S.J. Etuk, F. Duke, Staff attitude as barrier to the utilization of University of Calabar Teaching Hospital for Obstetric care. African Journal of Reproductive Health 2000. 4(2): p. 69-73.

7. Atuyambe, L., F. Mirembe, J. Annika, E.K. Kirumira, E. Faxelid, Seeking safety and empathy: adolescent health seeking behavior during pregnancy and early motherhood in central Uganda. Journal of adolescence, 2009. 32(4): p. 781-96.

8. Avortri, G.S., A. Beke, G. Abekah-Nkrumah, Predictors of satisfaction with child birth services in public hospitals in Ghana. International journal of health care quality assurance, 2011. 24(3): p. 223-37.

9. Boller, C., K. Wyss, D. Mtasiwa, M. Tanner, Quality and comparison of antenatal care in public and private providers in the United Republic of Tanzania. Bulletin of the World Health Organization, 2003. 81(2): p. 116-22.

10. Brookes, H.B., Experiences of childbirth in Natal Indian Women. Curationis, 1991. 14(4): p. 4-9.

11. Campero, L., C. Garcia, C. Diaz, O. Ortiz, S. Reynoso, A. Langer, "Alone, I wouldn't have known what to do": a qualitative study on social support during labor and delivery in Mexico. Social science & medicine, 1998. 47(3): p. 395-403.

12. Chadwick, R.J., D. Cooper, J. Harries, Narratives of distress about birth in South African public maternity settings: A qualitative study. Midwifery, 2014. 30: p. 862 - 868.

13. Cham, M., J. Sundby, S. Vangen, Maternal mortality in the rural Gambia, a qualitative study on access to emergency obstetric care. Reproductive health, 2005. 2(1): p. 3.

14. Chapman, R.R., Endangering safe motherhood in Mozambique: prenatal care as pregnancy risk. Social science & medicine, 2003. 57(2): p. 355-74.

15. D'Ambruoso, L., M. Abbey, J. Hussein, Please understand when I cry out in pain: women's accounts of maternity services during labour and delivery in Ghana. BMC public health, 2005. 5: p. 140.

16. Duong, D.V., C.W. Binns, A.H. Lee, Utilization of delivery services at the primary health care level

in rural Vietnam. Social Science & Medicine, 2004. 59: p. 2585 - 2595.

17. Foster, J., R. Burgos, C. Tejada, R. Caceres, A.T. Altamonte, L.J. Perez, et al., A community-based participatory research approach to explore community perceptions of the quality of maternal-newborn health services in the Dominican Republic. Midwifery, 2010. 26(5): p. 504-11.

18. Gao, Y., L. Barclay, S. Kildea, M. Hao, S. Belton, Barriers to increasing hospital birth rates in rural Shanxi Province, China. Reproductive health matters, 2010. 18(36): p. 35-45.

19. Gebrehiwot, T., I. Goicolea, K. Edin, M. San Sebastian, Making pragmatic choices: women’s experiences of delivery care in Northern Ethiopia. BMC Pregnancy & Childbirth, 2012. 12: 113.

20. Gilson, L., H. Kitange, T. Teuscher, Assessment of process quality in Tanzanian primary care. Health policy, 1993. 26(2): p. 119-39.

21. Grossmann-Kendall, F., V. Filippi, M. De Koninck, L. Kanhonou, Giving birth in maternity hospitals in Benin: testimonies of women. Reproductive health matters, 2001. 9(18): p. 90-8.

22. Hadwiger, M.C., S.C. Hadwiger, Filipina mothers’ perceptions about childbirth at home. International Nursing Review, 2012. 59: p 125 - 131.

23. Hassan-Bitar, S., S. Narrainen, 'Shedding light' on the challenges faced by Palestinian maternal health-care providers. Midwifery, 2011. 27(2): p. 154-9.

24. Igboanugo, G.M., C.H. Martin, What are pregnant women in a rural Niger Delta community's perceptions of conventional maternity service provision? An exploratory qualitative study. African journal of reproductive health, 2011. 15(3): p. 59-72.

25. Ith, P., A. Dawson, C.S. Homer, Women's perspective of maternity care in Cambodia. Women and birth : journal of the Australian College of Midwives, 2013. 26(1): p. 71-5.

26. Izugbara, C., A. Ezeh, J.C. Fotso, The persistence and challenges of homebirths: perspectives of traditional birth attendants in urban Kenya. Health policy and planning, 2009. 24(1): p. 36-45.

27. Jaffre, Y., A. Prual, Midwives in Niger: an uncomfortable position between social behaviours and health care constraints. Social science & medicine, 1994. 38(8): p. 1069-73.

28. Jewkes, R., N. Abrahams, Z. Mvo, Why do nurses abuse patients? Reflections from South African obstetric services. Social science & medicine, 1998. 47(11): p. 1781-95.

29. Kaartinen, L., V. Diwan, Mother and child health care in Kabul, Afghanistan with focus on the mother: women's own perspective. Acta obstetricia et gynecologica Scandinavica, 2002. 81(6): p. 491-501.

30. Kabakian-Khasholian, T., O. Campbell, M. Shediac-Rizkallah, F. Ghorayeb, Women's experiences of maternity care: satisfaction or passivity? Social science & medicine, 2000. 51(1): p. 103-13.

31. Kabali, E., C. Gourbin, V. De Brouwere, Complications of childbirth and maternal deaths in Kinshasa hospitals: testimonies from women and their families. BMC pregnancy and childbirth, 2011. 11: p. 29.

32. Kempe, A., F.A. Noor-Aldin Alwazer, T. Theorell, Women's authority during childbirth and Safe Motherhood in Yemen. Sexual & reproductive healthcare : official journal of the Swedish Association of Midwives, 2010. 1(4): p. 129-34.

33. Kowalewski, M., A. Jahn, S.S. Kimatta, Why do at-risk mothers fail to reach referral level? Barriers beyond distance and cost. African journal of reproductive health, 2000. 4(1): p. 100-9.

34. Kruk, M.E., S. Kujawski, G. Mbaruku, K. Ramsey, W. Moyo, L.P. Freedman, Disrespectful and abusive treatment during facility delivery in Tanzania: a facility and community survey. Health Policy and Planning, 2014. doi: 10.1093/heapol/czu079

35. Kumbani, L., G. Bjune, E. Chirwa, A. Malata, J. Øyvind Odland, Why some women fail to give birth at health facilities: a qualitative study of women’s perceptions of perinatal care from rural Southern Malawi, 2013. Reproductive Health, 2013. 10:9.

36 Kyomuhendo, G.B., Low use of rural maternity services in Uganda: impact of women's status, traditional beliefs and limited resources. Reproductive health matters, 2003. 11(21): p. 16-26.

37. Langer, A., G. Nigenda, M. Romero, G. Rojas, C. Kuchaisit, M. al-Osimi, et al., Conceptual bases and methodology for the evaluation of women's and providers' perception of the quality of antenatal care in the WHO Antenatal Care Randomised Controlled Trial. Paediatric and perinatal epidemiology, 1998. 12 Suppl 2: p. 98-115.

38. Larsen, G.L., S. Lupiwa, H.P. Kave, S. Gillieatt, M.P. Alpers, Antenatal care in Goroka: issues and perceptions. Papua and New Guinea medical journal, 2004. 47(3-4): p. 202-14.

39. Lubbock, L.A., R.B. Stephenson, Utilization of maternal health care services in the department of Matagalpa, Nicaragua. Revista panamericana de salud publica = Pan American journal of public health, 2008. 24(2): p. 75-84.

40. MacKeith, N., O.J. Chinganya, Y. Ahmed, S.F. Murray, Zambian women's experiences of urban maternity care: results from a community survey in Lusaka. African journal of reproductive health, 2003. 7(1): p. 92-102.

41. Magoma, M., J. Requejo, O.M.R. Campbell, S. Cousens, V. Filippi, High ANC coverage and low skilled attendance in a rural Tanzanian district: a case for implementing a birth plan intervention. BMC Pregnancy & Childbirth, 2010. 10: 13.

42. Mathole, T., G. Lindmark, F. Majoko, B.M. Ahlberg, A qualitative study of women's perspectives of antenatal care in a rural area of Zimbabwe. Midwifery, 2004. 20(2): p. 122-32.

43. McMahon, S.A., A.S. George, J.J. Chebet, I.H. Mosha, R.N.M. Mpembeni, P.J. WInch, Experiences of and responses to disrespectful maternity care and abuse during childbirth; a qualitative study with women and men in Morogoro Region, Tanzania. BMC Pregnancy & Childbirth, 2014. 14: 268.

44 Medeiros, A.L., L.P. Landim, M.C.M. Sousa, R.W. de Lima Cabral, S. Ribeiro Santos, Interpersonal Relations among Professional Care and Women With Experience of Abortion in Hospital Environment. Journal of Nursing UFPE Online 2013. 7(2): p. 452-9.

45 Miller, S., M. Cordero, A.L. Coleman, J. Figueroa, S. Brito-Anderson, R. Dabagh, et al., Quality of care in institutionalized deliveries: the paradox of the Dominican Republic. International journal of gynaecology and obstetrics: the official organ of the International Federation of Gynaecology and Obstetrics, 2003. 82(1): p. 89-103; discussion 87-8.

46. Moore, M., R. Copeland, I. Chege, D. Pido, M. Griffiths, A behaviour change approach to investigating factors influencing women's use of skilled care in Homa Bay District, Kenya. Working draft. 2002, Academy for Educational Development: CHANGE Project: Washington, D.C.

47. Mrisho, M., J.A. Schellenberg, A.K. Mushi, B. Obrist, H. Mshinda, M. Tanner, et al., Factors affecting home delivery in rural Tanzania. Tropical medicine & international health : TM & IH, 2007. 12(7): p. 862-72.

48. Mselle, L.T., T.W. Kohi, A. Mvungi, B. Evjen-Olsen, K.M. Moland, Waiting for attention and care: birthing accounts of women in rural Tanzania who developed obstetric fistula as an outcome of labour. BMC pregnancy and childbirth, 2011. 11: p. 75.

49. Mumtaz, Z., S. Salway, M. Waseem, N. Umer, Gender-based barriers to primary health care provision in Pakistan: the experience of female providers. Health policy and planning, 2003. 18(3): p. 261-9.

50. Murira, N., K. Lützen, G. Lindmark, K. Christensson, Communication patterns between health care providers and their clients an antenatal clinic in Zimbabwe**.** Health Care for Women International, 2003. 24: p. 83 - 92.

51. Mwangome, F.K., P.A. Holding, K.M. Songola, G.K. Bomu, Barriers to hospital delivery in a rural setting in Coast Province, Kenya: community attitude and behaviours. Rural and remote health, 2012. 12: p. 1852.

52. Nabukera, S.K., K. Witte, C. Muchunguzi, F. Bajunirwe, V.K. Batwala, E.M. Mulogo, et al., Use of postpartum health services in rural Uganda: knowledge, attitudes, and barriers. Journal of community health, 2006. 31(2): p. 84-93.

53. Ngomane, S., F.M. Mulaudzi, Indigenous beliefs and practices that influence the delayed attendance of antenatal clinics by women in the Bohlabelo district in Limpopo, South Africa. Midwifery, 2012. 28(1): p. 30-8.

54. Nguyen, M.H., T. Gammeltoft, V. Rasch, Situation analysis of quality of abortion care in the main maternity hospital in Hai Phong, Viet Nam. Reproductive health matters, 2007. 15(29): p. 172-82.

55. Nigenda, G., A. Langer, C. Kuchaisit, M. Romero, G. Rojas, M. Al-Osimy, et al., Womens' opinions on antenatal care in developing countries: results of a study in Cuba, Thailand, Saudi Arabia and Argentina. BMC public health, 2003. 3: p. 17.

56. Okafor, C.B., R.R. Rizzuto, Women's and health-care providers' views of maternal practices and services in rural Nigeria. Studies in family planning, 1994. 25(6 Pt 1): p. 353-61.

57. Onah, H.E., L.C. Ikeako, G.C. Iloabachie, Factors associated with the use of maternity services in Enugu, southeastern Nigeria. Social science & medicine, 2006. 63(7): p. 1870-78.

58. Otis K.E.,J.A. Brett, Barriers to hospital births: why do many Bolivian women give birth at home? Rev Panam Salud Publica, 2008. 24(1): p 46–53.

59. Oyerinde K., Y. Harding • P. Amara, N. Garbrah-Aidoo, R. Kanu, M. Oulare, R. Shoo, K. Daoh, A Qualitative Evaluation of the Choice of Traditional Birth Attendants for Maternity Care in 2008 Sierra Leone: Implications for Universal Skilled Attendance at Delivery. Maternal Child Health Journal 2013. 17: p 862 - 868.

60. Pell, C., A. Menaca, F. Were, N.A. Afrah, S. Chatio, L. Manda-Taylor, et al., Factors affecting antenatal care attendance: results from qualitative studies in Ghana, Kenya and Malawi. PloS one, 2013. 8(1): p. e53747.

61. Pettersson, K.O., E. Johansson, F. Pelembe Mde, C. Dgedge, K. Christensson, Mozambican midwives' views on barriers to quality perinatal care. Health care for women international, 2006. 27(2): p. 145-68.

62. Pretorius, C.F., M. Greeff, Health-service utilization by pregnant women in the greater Mafikeng-Mmabatho district. Curationis, 2004. 27(1): p. 72-81.

63. Prytherch, H., M. Kagone, G.A. Aninanya, J.E. Williams, D.C. Kakoko, M.T. Leshabari, et al., Motivation and incentives of rural maternal and neonatal health care providers: a comparison of qualitative findings from Burkina Faso, Ghana and Tanzania. BMC health services research, 2013. 13: p. 149.

64. Rahmani, Z., M. Brekke, Antenatal and obstetric care in Afghanistan--a qualitative study among health care receivers and health care providers. BMC health services research, 2013. 13: p. 166.

65. Rai, S.K., R. Dasgupta, M.K. Das, S. Singh, R. Devi, N.K. Arora, Determinants of utilization of services under MMJSSA scheme in Jharkhand 'Client Perspective': a qualitative study in a low performing state of India. Indian journal of public health, 2011. 55(4): p. 252-9.

66. Ruiz, M.J., M.G. van Dijk, K. Berdichevsky, A. Munguia, C. Burks, S.G. Garcia, Barriers to the use of maternity waiting homes in indigenous regions of Guatemala: a study of users' and community members' perceptions. Culture, health & sexuality, 2013. 15(2): p. 205-18.

67. Seljeskog, L., J. Sundby, J. Chimango, Factors influencing women's choice of place of delivery in rural Malawi--an explorative study. African journal of reproductive health, 2006. 10(3): p. 66-75.

68. Shiferaw, S., M. Spigt, M. Godefrooij, Y. Melkamu, M. Tekie, Why do women prefer home births in Ethiopia? BMC pregnancy and childbirth, 2013. 13: p. 5.

69. Silal, S., L. Penn-Kekana, B. Harris, S. Birch, D. McIntyre, Exploring inequalities in access to and use of maternal health services in South Africa. BMC Health Services Research, 2012. 12: 120.

70. Smith, E., S.F. Murray, A.K. Yousafzai, L. Kasonka, Barriers to accessing safe motherhood and reproductive health services: the situation of women with disabilities in Lusaka, Zambia. Disability and rehabilitation, 2004. 26(2): p. 121-7.

71 Solo, J., Easing the pain: pain management in the treatment of incomplete abortion. Reproductive health matters, 2000. 8(15): p. 45-51.

72. Spangler, S.A, S.S. Bloom, Use of biomedical obstetric care in rural Tanzania: The role of social and

material inequalities. Social Science & Medicine, 2010. 71: p 760 - 768.

73. Tilahun, M., B. Mengistie, G. Egata, A.A. Reda, Health workers' attitudes toward sexual and reproductive health services for unmarried adolescents in Ethiopia. Reproductive health, 2012. 9: p. 19.

74. Titaley, C.R., C.L. Hunter, M.J. Dibley, P. Heywood, Why do some women still prefer traditional birth attendants and home delivery?: a qualitative study on delivery care services in West Java Province, Indonesia. BMC pregnancy and childbirth, 2010. 10: p. 43.

75. Tlebere, P., D. Jackson, M. Loveday, L. Matizirofa, N. Mbombo, T. Doherty, et al., Community-based situation analysis of maternal and neonatal care in South Africa to explore factors that impact utilization of maternal health services. Journal of midwifery & women's health, 2007. 52(4): p. 342-50.

76. Tuncalp, O., M.J. Hindin, K. Adu-Bonsaffoh, R. Adanu, Listening to Women's Voices: The Quality of Care of Women Experiencing Severe Maternal Morbidity, in Accra, Ghana. PLOS One, 2012. 7(8): e44536.

77. Uzochukwu, B.S., O.E. Onwujekwe, C.O. Akpala, Community satisfaction with the quality of maternal and child health services in southeast Nigeria. East African medical journal, 2004. 81(6): p. 293-9.

78. Vera, H., The client's view of high-quality care in Santiago, Chile. Studies in family planning, 1993. 24(1): p. 40-9.

79. Weeks, A., T. Lavender, E. Nazziwa, F. Mirembe, Personal accounts of 'near-miss' maternal mortalities in Kampala, Uganda. BJOG : an international journal of obstetrics and gynaecology, 2005. 112(9): p. 1302-7.

80. Wild, K., L. Barclay, P. Kelly, N. Martins, Birth choices in Timor-Leste: A framework for understanding the use of maternal health services in low resource settings. Social Science & Medicine, 2010. 71: p 2038 - 2045.

81. Yakong, V.N., K.L. Rush, J. Bassett-Smith, J.L. Bottorff, C. Robinson, Women's experiences of seeking reproductive health care in rural Ghana: challenges for maternal health service utilization. Journal of advanced nursing, 2010. 66(11): p. 2431-41.
