# Supplementary material for: Attitudes and behaviours of maternal health care providers in interactions with clients: a systematic review
Source: Global Health. 2015 Aug 15;11:36. doi: 10.1186/s12992-015-0117-9 (PMC4537564; doi:10.1186/s12992-015-0117-9)
Supplement: Supplementary file 2 — Characteristics and findings of included studies. [file 12992_2015_117_MOESM2_ESM.docx]

**SUPPLEMENTARY MATERIAL: SEARCH STRATEGIES**

**Search strategy used in PubMed**

(Maternal) AND (Health personnel OR health worker* OR healthcare worker* OR health care worker* OR doctor OR doctors OR physician* OR nurse* OR midwife OR midwives OR gynaecologist OR gynecologist* OR obstetrician* OR health practitioner* OR traditional birth attendant* OR birt**h** attendant*))

AND

((Behaviour* OR behavior* OR intention* OR practice* OR attitude* OR belief * OR idea* OR Attitude of Health Personnel[majr] OR health knowledge, attitudes, practice[majr] OR delivery of health care/trends[mh]))

AND

((Factor OR factors OR Barrier* OR challenge* OR deterrent* OR facilitator* OR opportunit* OR improve) AND (Patient OR patients OR client* OR mother* OR woman OR women) AND (patient acceptance of health care[mh] OR patient satisfaction[mh] OR acceptability OR health seeking behavior* OR health seeking behaviour* OR care seeking OR attendance OR service utilization OR service utilisation OR service use OR perception* OR perspective* OR experience OR Access to service* OR provision of service* OR delivery of service*))

AND

developing country[mh]

Filters: Publication date from 1990/01/01 to 2013/06/01

**Search strategy used in CINAHL and PsycInfo**

(maternal) AND (Health personnel OR health worker* OR healthcare worker* OR health care worker* OR doctor OR doctors OR physician* OR nurse* OR midwife OR midwives OR gynaecologist* OR gynecologist* OR obstetrician* OR health practitioner* OR traditional birth attendant* OR birth attendant*)

AND

Behaviour* OR behavior* OR intention* OR practice* OR attitude* OR belief * OR idea*

AND

Factor OR factors OR Barrier* OR challenge* OR deterrent* OR facilitator* OR opportunit* OR improve

AND

Patient* OR client* OR mother* OR woman OR women

AND

satisfaction OR acceptability OR health seeking behavior* OR health seeking behaviour* OR care seeking OR attendance OR service utilization OR service utilisation OR service use OR perception OR perspective* OR experience* OR Access to service* OR provision of service* OR delivery of service*

AND

developing countries (subject word in CINAHL)

Limits: 1990 to 2013

**Search strategy used in Popline**

(Maternal AND Health personnel OR health worker* OR healthcare worker* OR health care worker* OR doctor OR doctors OR physician* OR nurse* OR midwife OR midwives OR gynaecologist* OR gynecologist* OR obstetrician* OR health practitioner* OR traditional birth attendant* OR birth attendant*)

AND

(Behaviour* OR behaviour* OR intention* OR practice* OR attitude* OR belief * OR idea*)

AND

Factor OR factors OR Barrier* OR challenge* OR deterrent* OR facilitator* OR opportunit* OR improve

AND

Developing countr*

Limits: 1990 to 2013

**Search strategy used in the Cochrane Library**

Maternal AND health worker AND attitude OR behaviour
